# Resting state EEG biomarkers of cognitive decline associated with Alzheimer’s disease and mild cognitive impairment

**DOI:** 10.1371/journal.pone.0244180

**Published:** 2021-02-05

**Authors:** Amir H. Meghdadi, Marija Stevanović Karić, Marissa McConnell, Greg Rupp, Christian Richard, Joanne Hamilton, David Salat, Chris Berka

**Affiliations:** 1 Advanced Brain Monitoring Inc., Carlsbad, CA, United States of America; 2 University of California, San Diego, San Diego, CA, United States of America; 3 Advanced Neurobehavioral Health of Southern California, San Diego, CA, United States of America; 4 Massachusetts General Hospital, Boston, MA, United States of America; 5 Harvard Medical School, Boston, MA, United States of America; Nathan S Kline Institute, UNITED STATES

## Abstract

In this paper, we explore the utility of resting-state EEG measures as potential biomarkers for the detection and assessment of cognitive decline in mild cognitive impairment (MCI) and Alzheimer’s disease (AD). Neurophysiological biomarkers of AD derived from EEG and FDG-PET, once characterized and validated, would expand the set of existing diagnostic molecular biomarkers of AD pathology with associated biomarkers of disease progression and neural dysfunction. Since symptoms of AD often begin to appear later in life, successful identification of EEG-based biomarkers must account for age-related neurophysiological changes that occur even in healthy individuals. To this end, we collected EEG data from individuals with AD (n = 26), MCI (n = 53), and cognitively normal healthy controls stratified by age into three groups: 18–40 (n = 129), 40–60 (n = 62) and 60–90 (= 55) years old. For each participant, we computed power spectral density at each channel and spectral coherence between pairs of channels. Compared to age matched controls, in the AD group, we found increases in both spectral power and coherence at the slower frequencies (Delta, Theta). A smaller but significant increase in power of slow frequencies was observed for the MCI group, localized to temporal areas. These effects on slow frequency spectral power opposed that of normal aging observed by a decrease in the power of slow frequencies in our control groups. The AD group showed a significant decrease in the spectral power and coherence in the Alpha band consistent with the same effect in normal aging. However, the MCI group did not show any significant change in the Alpha band. Overall, Theta to Alpha ratio (TAR) provided the largest and most significant differences between the AD group and controls. However, differences in the MCI group remained small and localized. We proposed a novel method to quantify these small differences between Theta and Alpha bands’ power using empirically derived distributions of spectral power across the time domain as opposed to averaging power across time. We defined Power Distribution Distance Measure (PDDM) as a distance measure between probability distribution functions (pdf) of Theta and Alpha power. Compared to average TAR, using PDDF enhanced the statistical significance, the effect size, and the spatial distribution of significant effects in the MCI group. We designed classifiers for differentiating individual MCI and AD participants from age-matched controls. The classification performance measured by the area under ROC curve after cross-validation were AUC = 0.85 and AUC = 0.6, for AD and MCI classifiers, respectively. Posterior probability of AD, TAR, and the proposed PDDM measure were all significantly correlated with MMSE score and neuropsychological tests in the AD group.

## Introduction

Worldwide, the prevalence of Alzheimer’s disease (AD) is rapidly increasing [1,2] with no approved disease modifying treatment [3]. Pathophysiological neural processes underlying the Alzheimer’s disease are believed to precede the overt presentation of clinical symptoms by decades [4,5]. Therefore, there is an important need for biomarkers of Alzheimer’s disease to characterize and monitor both natural progression of the disease and potential therapeutic interventions. Recent clinical guidelines focus on a “biological rather than syndromal” definition of AD [6] particularly because AD is likely a gradual accumulation of multiple pathologies [7]. Therefore, pathological biomarkers of AD are widely used in clinical practice. These biomarkers include cerebrospinal fluid (CSF) beta amyloid deposition and pathologic tau as well as imaging methods that identify neurodegeneration such as magnetic resonance imaging (MRI) and fluorodeoxyglucose (FDG) PET. Such diagnostic biomarkers directly reflect the pathophysiological basis of AD and hence are essential in diagnosis and characterization of AD [6,8–10]. However, to better understand the disease progression, both pathology and the resulting syndromes should be studied. Pathology leads to impaired neural functions, which in turn leads into clinical syndromes such as cognitive decline/dementia. This is a complicated process and AD progression in its most common form starts with preclinical asymptomatic stage into mild cognitive impairment (MCI) and finally overt dementia and severe impairment. Current standard of clinical practice relies on CSF and imaging biomarkers to identify the pathological changes in the brain as the cause while using neuropsychological tests to assess the clinical syndromes as an effect or outcome of the disease. CSF and imaging biomarkers are expensive and/or invasive and neuropsychological tests are not sensitive to subtle changes in cognition.

**Fig 1:**
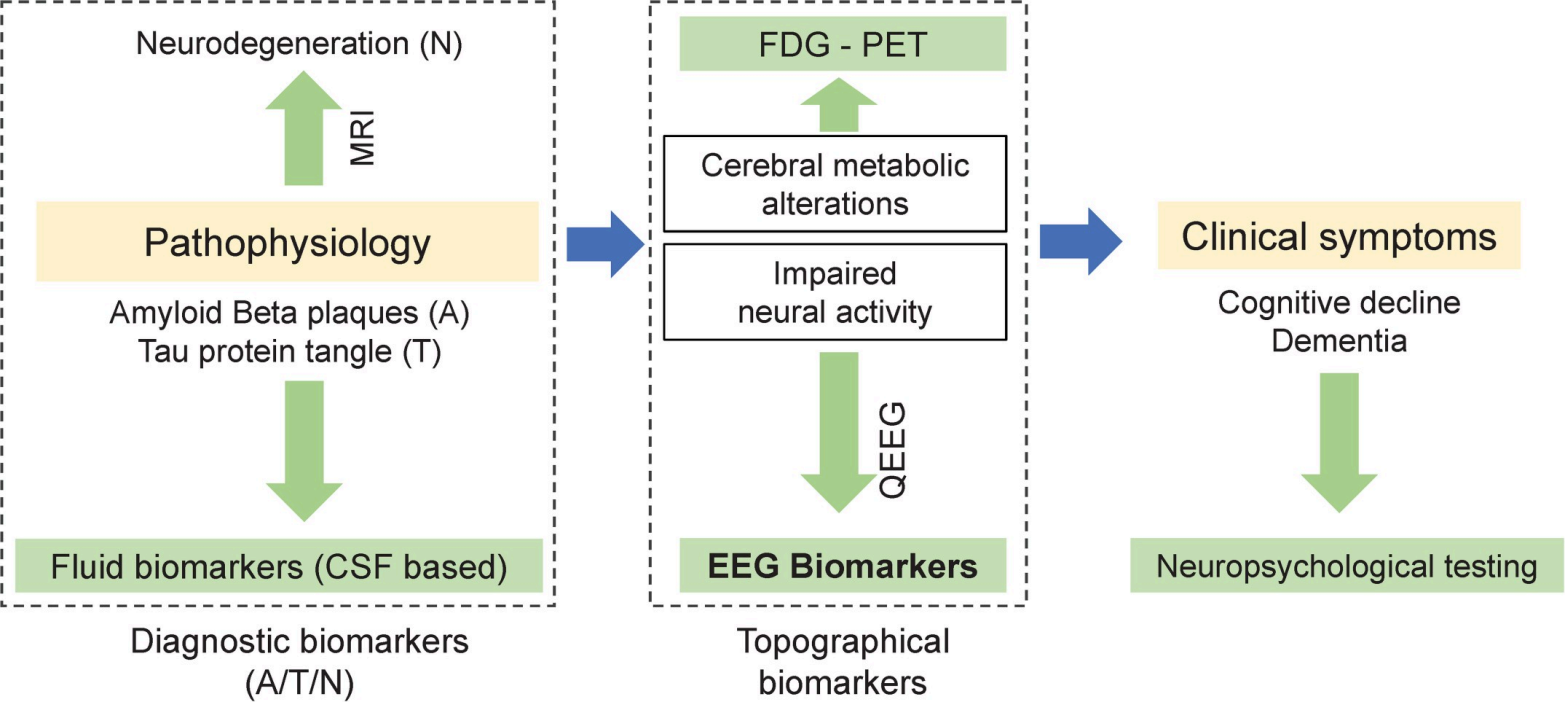
EEG as a biomarker: While Diagnostic biomarkers of Alzheimer’s disease (left) are linked to pathophysiology, topographical biomarkers (middle) in general and EEG-biomarkers in particular, can be linked to impaired neural activities that are the basis of impaired cognitive processes.

A complimentary approach is using electroencephalography (EEG) to derive neurophysiological measures associated with neural activities that are the basis of cognitive processes (Fig 1). EEG recorded at the scalp can reflect subtle functional changes in the cortex (Nunez et al. 2006). Although EEG and other topographical biomarkers may not be used as definitive diagnostic tools, they have been shown to be associated with abnormalities and progression of AD [11–19]. A growing body of evidence suggests that EEG biomarkers can be used to identify early stage abnormalities in neuronal function before measurable cortical tissue loss or cognitive decline [12,14,20–24].

Unlike other methods of acquiring candidate AD biomarkers, EEG provides a noninvasive and relatively inexpensive measure of brain activity with established utility [12,21,25]. Although the use of EEG to study the brain dates back to the 1930’s, the past decade has witnessed a resurgence of interest in EEG [26], likely due to increasing availability of analytical techniques and computational power for quantifying EEG patterns beyond what is recognizable by visual inspection. EEG can be recorded while subjects are engaged in various cognitive tasks to identify cognitive processes that may be characteristically perturbed in individuals with AD or MCI (e.g. [27]). However, the simplest and most common manner of acquiring EEG is recording background activity while the subject is in a resting state with their eyes open (EO) or eyes closed (EC). Analysis of the power spectral density (PSD) is the most common method of quantifying EEG patterns. According to recent reviews [11,12,14,28], the most commonly reported resting-state EEG findings that distinguish participants with AD or MCI from unimpaired control subjects are diffused slowing of the EEG i.e. increased power in lower frequency bands. Specifically, progression to AD is characterized by increasing Delta and Theta power accompanied by decreasing Alpha and Beta power [20,29–37]). The ratio of power at different frequency bands have also been defined as such characterizing EEG biomarkers [38].

Other analysis techniques such as coherence, causality and functional coupling and synchrony across cortical regions have also been used to detect abnormalities in AD [39–43]. Several studies reported changes in EEG coherence in AD, specifically reduction in Alpha coherence [24,25,39,43,44]. EEG biomarkers of AD have also been linked to other biomarkers including reductions of glucose metabolism in the temporo-parietal regions as measured by FDG PET [45], cortical and hippocampal atrophy measured with structural MRI, decreased levels of beta amyloid, and increased levels of phospho-tau and total tau in the CSF [46].

Unlike AD, EEG abnormalities in MCI are smaller and have not been consistently replicated, likely as a result of the heterogeneity of the MCI population [47]. However, there is preliminary evidence from longitudinal studies that the progression from MCI to AD may be associated with several of these EEG biomarkers [27,47–50]).

Importantly, significant changes in the power spectral characteristics of the resting state EEG also occur as a result of normal aging [51–53]. These changes have been reported as a progressive diminution in spectral power at Delta and Theta frequency bands as well as reduction and slowing of the posterior dominant rhythm in alpha band. Thus, the changes in Delta and Theta power due to AD are in direct contrast with those associated with healthy aging.

The overall goal of the current study was to identify and validate EEG-derived measures of cognitive decline caused by AD or MCI. The preliminary findings and a subset of these biomarkers (based on average power spectral densities across recording sessions) were previously reported in [17] using a smaller subset of participants. The specific purpose and the contribution of the current manuscript is twofold: (1) to improve sensitivity of EEG-based biomarkers of AD by introducing EEG measures that better identify mild cognitive impairment (MCI), and (2) to compare EEG changes due to cognitive decline as opposed to healthy aging by comparing similarities and differences between the two in terms of their effect on EEG biomarkers.

Due to nonstationary nature of EEG time series, the most common method to define PSD-based EEG biomarkers is to quantify EEG patterns at short time intervals (epochs) and to average the EEG measures across epochs. We hypothesized that EEG abnormalities in MCI are not only smaller in size but likely less frequent during a recording session. Therefore, we proposed a novel method based on the statistical distribution of quantified EEG measures across a session, i.e. the empirical probability distribution function. We implemented our standard and modified EEG biomarkers and identified significant differences between cognitively impaired AD or MCI groups compared to unimpaired controls in different age groups. We validated the biomarkers by (a) evaluating classification algorithms that separate MCI and AD groups from age matched controls and (b) identifying the correlation between the proposed EEG biomarkers and clinical measures of cognitive decline obtained by neuropsychological testing. We also compared participant groups based on spectral coherence measures and identified the most significant frequency bands that are affected by normal aging, MCI and AD.

## Materials and methods

### Participants

Volunteer individuals between the ages of 18–90 were recruited across four sites in the United Sates as described below. Advanced Brain Monitoring (ABM) in Carlsbad, California, USA; Advanced Neurobehavioral Health (ANH) in San Diego, California, USA; Massachusetts General Hospital (MGH) in Boston, Massachusetts, USA; and Mayo clinic (MAYO) in Rochester, Minnesota, USA. Table 1 summarizes the participants’ demographic information. There were no specific requirements for gender, race, or ethnicity. All participants were selected after the successful completion of both a screening interview by telephone, and a comprehensive in-office clinical and personal information questionnaire. Institutional Review Boards (IRB) at each site approved the study protocol (Advarra CIRBI at ABM and ANH sites, Mass General Brigham Human Research Committee at MGH site, and Institutional Review Board at Mayo Clinic at MAYO site). EEG acquisitions were scheduled in the mornings and participants were instructed to refrain from caffeine before the experiment. The time of the acquisition during the day is reported in Table 1. All participants provided informed consent. The capacity to provide consent was implied after the screening interview and it was not formally assessed. Assessment was not needed because only healthy participants or participants with mild cognitive impairment or mild dementia were recruited for the study. For MCI and AD participants, the consent procedure was reviewed with the patients’ care partner present.

**Table 1:** Participant groups and demographic information.

	HC1 (18–40)	HC2 (40–60)	HC3 (60–90)	MCI	AD
N	129	62	55	53	26
Study Site	ABM (n = 129)	ABM (n = 61) MGH (n = 1)	ABM (n = 43) MGH (n = 12)	MGH (n = 19) ANH (n = 27) MAYO (n = 7)	ANH (n = 26)
Age range	18–38	40–60	61–84	53–88	58–90
Age (mean ± SD)	24.4 ± 4.3	50.9 ± 6.6	68.8 ± 5.7	71.2 ± 7.8	73.5 ± 8.5
% Female	53%	52%	45%	38%	50%
MMSE (mean ± SD)	NA	29.8 ± 0.8	29.7 ± 0.6	26.8 ± 2.8	18.58 ± 5.9
MMSE (median)	NA	30	30	27	19
Beck Depression Inventory BDI II (mean ± SD)	6.0±6.8	3±4.6 (n = 59)	2.7±4.0	5.6±5.7	5.0±5.57
BDI II (median)	4	1 (n = 59)	2	4	2.5
Mayo Fluctuation	NA	0.52 ± 0.8	0.46 ± 0.7	0.68 ± 0.9	0.8 ± 1.0
Clinical Assessment of Fluctuation %nonzero (CAF/ODF)	NA	10% (0.06/0.1)	14% (0.05/0.26)	28% (0.95/0.5)	53% (1.69,1.42)
% with Diabetes	0%	3%	9%	10%	12%
% with Heart Disease	0%	0%	9%	13%	23%
% with High Blood Pressure	0%	18%	42%	28%	31%
Education Level (university/high school)	38%, 98%	43%, 98%	* 50%, 96%	58%, 98%	54%, 96%
Handedness %RH/ %LH	94/5	89/8	91/6	73/13	88/12
Pareidolia Test (%correct, %false positive)	N/A	100, 0 (n = 59)	99, 0 (n = 55)	96, 3 (n = 48)	84,11 (n = 20)
EEG Acquisition, time of the day (Hours)	10.1±0.8	10.9±1.8	11.3±2.1	11.6±1.6	12±1.2

Missing data note: If less than 98% of the data for each participant group are available, the number of available records is shown in paratheses.

#### Healthy controls

Cognitively normal participants in three age groups [between the ages of 18–39 (HC1, n = 129), 40–60 (HC2, n = 62) and 60–90 (HC3, n = 52)] were recruited by Advanced Brain Monitoring (ABM) in Carlsbad, California (n = 43) through online ads and flyers and by Massachusetts General Hospital MGH (n = 12) in Boston, Massachusetts. Participants in HC1 group were originally recruited for a different study but underwent the same recruitment and study protocol as other groups.

#### Individuals with Alzheimer’s disease (AD)

Participants in the AD group were recruited at Advanced Neurobehavioral Health (ANH) study site. AD group were recruited from the greater San Diego area from either 1) a pool of individuals who enrolled in the Shiley-Marcos University of California, San Diego (UCSD) Alzheimer’s Disease Research Center (ADRC) longitudinal study and agreed to be contacted by outside researchers, or 2) a pool of individuals who were treated by community neurologists and indicated interest in research opportunities. Diagnostic criteria for AD were based on the neuropsychological evaluation as follows: a) presence of objective cognitive impairment (≥1.5 standard deviations) in the memory domain plus at least one other cognitive domain (i.e., language, visuospatial skills, executive functions, and/or complex attention and processing speed), b) decline in activities of daily living due to cognitive impairment, and c) absence of other medical or mental disease that explained the syndrome. Eligible participants from the ADRC were diagnosed by two senior staff neurologists based on criteria developed by the National Institute of Neurological and Communicative Disorders and Stroke and the Alzheimer’s Disease and Related Disorders Association (NINCDS-ADRDA McKhann et al, 1984, and revised in 2011). The ADRC diagnostic procedure has been extensively documented (see, Salmon & Butters, 1992). Briefly, all ADRC volunteers received annual evaluations including neurological evaluation and extensive neuropsychological testing. The ADRC neuropsychological battery included tests of global abilities [Mini Mental State Examination (MMSE) and Mattis Dementia Rating Scale (DRS)], verbal and non-verbal memory [California Verbal Learning Test (CVLT), Wechsler Memory Scale-revised (WMS-R) Logical Memory, and Heaton-modified visual reproduction), language (Boston Naming Test (BNT), Wechsler Adult Intelligence Scale-revised (WAIS-R) Vocabulary, category and letter fluency), visuospatial skills (cube copy and modified parietal lobe battery), executive functions (Trails A and B, WAIS-R similarities and arithmetic), and attentional (WAIS-R digit span) abilities. Community volunteers were diagnosed with AD according to revised NINCDS-ADRDA and DMS-5 criteria for Major Neurocognitive Disorder (i.e., dementia) due to Alzheimer’s disease. Community volunteers underwent a comprehensive neuropsychological evaluation by a board-certified clinical neuropsychologist (JMH) utilizing a battery that included tests of global abilities (DRS), verbal and non-verbal memory (CVLT-II, WMS-IV Logical Memory, and Rey Complex Figure Test), expressive and receptive language (Neuropsychological Assessment Battery Naming, Boston Diagnostic Aphasia Examination Complex Ideation Test, animal and letter fluency), visuospatial skills (cube copy, clock drawing, interlocking pentagons, WAIS-IV block design, and Judgment of Line Orientation), executive functions (e.g. Trails A and B, WAIS-IV similarities, an appropriate version of Wisconsin Card Sorting Test), and attentional (WAIS-IV digit span, coding, and symbol search) abilities.

#### Individuals with Mild Cognitive Impairment (MCI)

Individuals with Mild Cognitive Impairment (MCI, n = 53) were recruited by Massachusetts General Hospital (MGH, n = 19) or Advanced Neurobehavioral Health (ANH, n = 27) or Mayo Clinic (MAYO, n = 7).

Volunteer participants at ANH diagnosed with mild cognitive impairment (MCI), amnestic type, underwent the same recruitment and diagnostic procedures as the AD group (see above). Diagnosis was based on neuropsychological testing and adhered to DSM-5 criteria for minor neurocognitive disorder (i.e., MCI). Specifically, diagnosis criteria included: a) presence of objective cognitive impairment (≥1.5 standard deviation (SD)) in the memory domain, b) absence of decline in activities of daily living, and c) absence of medical or mental disease that explained the syndrome. Absence of decline in activities of daily living was confirmed by a knowledgeable informant, typically a spouse or adult child.

Volunteer participants at MGH were drawn from a longitudinal cohort at a major academic medical center for a study of brain aging and cognition. Participants were recruited through various sources including the affiliated hospitals and other local advertisements. Participants were generally neurologically and psychiatrically healthy as determined by a medical screen and a neurological evaluation and exhibited broadly normal global cognitive functioning at the time of the assessment (MMSE: 24–30). Diagnosis of MCI was determined through neuropsychological evidence following the “comprehensive criteria” proposed in [54] further standardized to require at least two domains with two or more impaired scores (i.e. 1 standard deviation below normative mean). Specifically, all the participants were given a comprehensive neuropsychological evaluation battery containing 14 neuropsychological tests assessing global cognitive functioning, premorbid intelligence, and four specific cognitive domains. All tests were scored using standardized norms adjusted for demographic variables including age, sex and education level. Sixteen standardized performance scores from the ten tests assessing the four specific cognitive domains were used to make neuropsychological classification. Table 1 summarizes the participants demographic information.

#### Exclusion criteria

Participants were excluded if they reported any of the following conditions: known neurological or psychiatric disorders, cardiac arrhythmias, heart failure (e.g. myocardial infarction), epilepsy, HIV+ diagnosis, bipolar disorder, or major depression. Participants were not excluded for controlled hypertension, diabetes, high cholesterol, treated mild to moderate sleep apnea, or mild depression. Medical marijuana use was not a cause for excluding a participant, however, current usage amounts and frequency of use were documented. All participants enrolled in the study were asked to disclose all medications that they were currently on and the last time they took their medication. After arriving to the site, research technicians explained the study protocol to the participants and addressed any additional questions. Once participants indicated they had no further questions, they were asked to sign the IRB approved consent form. Four healthy controls were removed prior to completion of the study. Removal of three participants was based on one or more of the exclusion criteria, and one dropped out in the middle of the study.

### Clinical and neuropsychological assessments

All participants across all study sites were administered a clinical and personal information questionnaire as well as the following tests: Beck Depression Inventory-II (BDI-II), United Parkinson’s Disease Rating Scale (UPDRS) with Modified Hoehn and Yahr, Mini Mental State Examination (MMSE), Pareidolia Task-Short Form, Clinical Assessments of Fluctuations [55], Mayo Fluctuations Scale, a sleep questionnaire, and Neuropsychiatric Inventory questionnaire. AD and MCI participants were also administered Mattis Dementia Rating Scale-II (DRS-II). The Wechsler Memory Scale and Hopkins Verbal Learning Test was also administered for some of participants in the MCI and AD groups. MMSE and Pareidolia test results are shown in Table 1. DRS-II test results are shown in Table 2 for the MCI and AD group as well as healthy participants (n = 12) that were recruited at MGH. We reported Clinical Assessments of Fluctuations (CAF) and One Day Fluctuation (ODF) Assessments scores [55] in Table 1 as follows. Percentage of participants in each group with nonzero scores in either CAF or ODF score were reported and the average CAF and ODF for those participants were also reported separately. Medication information for all participant groups is listed in Table 3.

**Table 2:** Neuropsychological test results group average ± standard error of the mean for each group are shown.

Neuropsychological test*	AD (n = 20)	MCI (n = 42)	HC3 (n = 12)
DRS-Attention (ATT)	32.7 ± 1.6	36.3 ± 0.3	36.4 ± 0.2
DRS-Memory (MEM)	12.6 ± 1.2	21.5 ± 0.5	23.5 ± 0.4
DRS-Initiation Preservation (IP)	27 ± 2.2	35.2 ± 0.4	36.4 ± 0.4
DRS-Conceptualization (CON)	32.2 ± 2.2	37.1 ± 0.3	38.5 ± 0.2
DRS-Construction (CN)	5.3 ± 0.4	5.8 ± 0.1	6 ± 0

**Table 3:** Percentage of participants taking medication.

Medication category	HC2	HC3	MCI	AD
Cognition-enhancing*	-	-	24%	54%
Antihypertensives** (AHT) total	6%	20%	27%	15%
AHT-ACE inhibitor	3%	5%	9%	-
AHT-Angiotensin receptor blockers (ARBs)	2%	7%	10%	12%
AHT-Alpha blocker	-	-	6%	0%
AHT-Beta blocker	3%	5%	9%	8%
AHT-Calcium channel blocker	-	4%	7%	-
AHT-Diuretics	-	7%	11%	12%
Antidepressants	6%	7%	30%	38%
Anti-diabetic	3%	2%	7%	-
Anticonvulsant (not for epilepsy)	-	7%	2%	8%
Antipsychotic	-	-	-	4%
Benzodiazepines	-	-	4%	8%
Statin	5%	13%	36%	19%
Sedatives	-	-	2%	4%
Dopamine Agonist	-	-	-	4%
Hormones	3%	7%	9%	8%
Opioid Narcotics	-	-	4%	-

* AChE Inhibitors (Rivastigmine, Donepezil or Galantamine) or NMDAR antagonist (Memantine).

** (Angiotensin Receptor blockers (ARBs), ACE Inhibitors, Alpha blockers, Beta blockers, and/or Diuretics).

### Experimental protocol

EEG and ECG (electrocardiogram) were recorded using STAT™ X24 (Advanced Brain Monitoring, Carlsbad, CA), an FDA-cleared wireless EEG system. Twenty channels of EEG configured in the standard 10–20 montage were recorded with reference to linked mastoids. One channel of ECG was recorded with sensors placed on the right and left clavicles. STAT™ X24 EEG uses passive Ag/AgCl electrodes with flexible circuit cables printed on polyester strips. The sampling rate was 256 Hz using an amplifier with low and high cutoff frequencies of 0.1 and 100 Hz, respectively. STAT™ X24 acquires high quality EEG without skin abrasion and uses a conductive cream to ensure good scalp interfaces (Kustomer Kinetics, Arcadia, CA). Technicians were instructed to keep the electrode impedances at or below 40kOhm [56] as per the manufacturer’s guideline.

EEG recordings were gathered during resting state using a structured testing and acquisition software platform with 5 minutes of eyes open and 5 minutes of eyes closed. The participants were seated in a comfortable chair with a laptop computer positioned on the desk directly in front of them. The laptop screen was situated approximately 65cm away from the participant’s face. During the eyes open task, participants were instructed to stare directly at a black fixation cross located in the center of a gray background. During the eyes closed task, they were instructed to close their eyes while maintaining wakefulness. After completion of the two resting state sessions, participants performed three event-related potential tasks (ERPs) that are outside of the scope of this paper.

### EEG power spectral analysis

EEG data during each session were imported into MATLAB (version R2017b). Data were bandpass filtered (1-49Hz) using a zero-phase Hamming windowed sinc FIR filter implemented in EEGLAB software v14.1.2 [57]. Invalid EEG channels with more than 5 seconds of flat line signal or having a correlation less than 0.4 with surrounding channel locations were excluded (less than 0.01% of total data were excluded) using clean_rawdata EEGLAB plugin v0.31. Independent component analysis (ICA) was performed using EEGLAB software [57]. ICLabel toolbox [58] v0.3.1 was used to automatically identify the source of independent components. ICLabel employs a classifier that is pretrained by thousands of labeled components obtained through crowdsourcing. Components classified as having sources other than brain (e.g. eye blinks, EMG, etc.) were automatically removed.

Power spectral densities (PSD) estimation of the clean EEG data were computed using LabX EEG processing software (Advanced Brain Monitoring Inc., Carlsbad, California). LabX uses modified periodogram PSD estimate with 1-second long Kaiser window (*b* = 6) and 50% overlap according to the following formula

$$psd=\frac{{2|X(f)|}^{2}}{f_{s}}$$ (1)

Where *f*_*s*_ = 256 is the sampling frequency and |*X*(*f*)|^2^ is the squared spectrum magnitude computed via Fast Fourier Transform (FFT, N = 256) of the windowed signal at each 1-Hz frequency bin, *f*, from 1 to 40 Hz. For each one-second non-overlapping epoch of the EEG signal at each channel, the average PSD of the 3 overlapped windowed signals were computed in a log-scale as $PSD=\log_{10}\left(\frac{{psd}_{k-1}+{psd}_{k}+{psd}_{k+1}}{3}\right)$ where *psd*_*k*_, *k* = 1,3,5,7,.. is the power spectral density estimate for the k^th^ windowed signal. A global PSD power was defined by averaging the PSDs across all 20 channels first and then converted into log-scale.

Absolute PSD for each 1-second epoch in each frequency bin from 1 to 60 Hz (1 Hz resolution) and each frequency bandwidth was computed using LabX. PSD in a frequency bandwidth is computed by averaging power across the frequency bins within its frequency range. Pre-defined bandwidths in LabX including Delta (1–3 Hz), Theta (3–7 Hz) and Alpha (8–13 Hz) were selected for further analysis. The full list of frequency bandwidths in LabX can be found in the supplementary section Table 1. We further analyzed the data in MATLAB as follows. Relative PSDs were computed by dividing the absolute PSD in each bin by the sum of the PSDs in 1–40 Hz frequency bins. Average PSDs at each channel for each participant were computed by averaging PSD values across epochs. Alpha peak frequency for each participant was computed by automatically finding the local peak in the average PSD within the frequency range (6–13 Hz). If more than one local peak was detected, the peak with the largest power was selected. PSD power at the alpha peak frequency was measured.

Distribution of power across epochs for each frequency band was computed. Group average PSD measures were computed for AD, MCI and the three healthy control groups. Only eyes-closed resting state data were selected for further analysis as our previous work (Meghdadi, 2019) and other reports [59] showed that overall, eyes closed EEG data better differentiate between AD participants and healthy controls.

### Analysis of coherence

EEG data were filtered, and artifacts were removed as per the methods in the previous section. Signals were segmented into 20-second epochs with 50% overlap. For each epoch and for each pair of EEG channels, the magnitude-square coherence estimate was computed using MATLAB (mscohere function) for each frequency bin. This function uses Welch’s overlapped average periodogram with a moving average 2-second Kaiser window and 50% overlap. For each participant and for each unique pairs of EEG channels, we defined a normalized coherence measure as follows. Normalized coherence was defined by subtracting the average of coherence values in frequency bins 1 to 40 Hz from the coherence measure in each frequency bin 1–40 Hz. Pairs of channels were divided into short-range or long-range based on the Euclidean distance between channel locations on a 2D map similar to the method described in [60]. Inter-hemispheric and intra-hemispheric long-range coherences were compared across AD, MCI and HC groups.

### Power distribution analysis

EEG biomarkers associated with AD include increased spectral power in lower frequencies, such as Delta and Theta and decreased power in Alpha band and higher frequencies, compared to healthy controls (Babiloni et al. 2019). However, the size of these spectral power effects, when averaged across the entire recording session, can be weak and are not consistently different in individuals with MCI, where changes in EEG may not be strong and widespread. Furthermore, it is conceivable that these changes may start with accumulative transient abnormalities that gradually progress into a consistent background pattern of activity. Therefore, analysis of the statistical distribution of EEG measures could be important in MCI. In this paper we proposed a novel method based on characterizing the distribution of EEG power at the epoch level within a session, rather than averaging power across the entire (5 minute) session. The method is fully described below.

For any given channel of EEG, data are divided into (N = 300) non-overlapping 1-second epochs {*e*_1_,…*e*_*N*_}. The average power spectral density of an individual epoch (in logarithmic scale) can be discretized by binning the power into M power bins *p*_1_,…,*p*_*M*_. Let *f*^*B*^(*p*_*k*_): 1≤*k*≤*M* be the normalized probability distribution function (PDF) of the EEG power at a given frequency band *B i*.*e*. *f*^*B*^(*p*_*k*_) represents the probability of a randomly selected epoch having a PSD power that falls in the frequency bin *p*_*k*_. Empirically, *f*^*B*^(*p*_*k*_) is estimated by computing the proportion of epochs for which the PSD power falls in the frequency bin *p*_*k*_ and is called empirical PDF. Furthermore, let $F^{B}(p_{k})=\sum_{j=1}^{k}f^{B}(p_{j})$ be the empirical cumulative distribution function (CDF) of power in bandwidth *B* such that *F*^*B*^(*p*_1_) = 0 and *F*^*B*^(*p*_*M)*_ = 1. ${F^{-1}}^{B}(u)$ is the inverse cumulative distribution function where 0<*u*<1 is the probability space and ${F^{-1}}^{B}(F^{B}(p_{k}))=1$.

We defined the power distribution difference function (PDDF) between any two bandwidths B_1_ and B_2_ as the difference between the corresponding inverse cumulative distribution functions as follows:

$${PDDF}_{{(B}_{1},B_{2})}(u)={F^{-1}}^{B_{1}}(u)-{F^{-1}}^{B_{2}}(u),0<u<1$$ (2)

Subsequently, a parametric power distribution distance measure (PDDM) between bandwidths *B*_*1*_ and *B*_*2*_ is defined by integrating PDDF over a given window of probabilities [*c*_1_ *c*_2_]:

$${PDDM}_{c_{1},c_{2}}(B_{1},B_{2})=\int_{c_{1}}^{c_{2}}{PDDF}_{{(B}_{1},B_{2})}(u)du$$ (3)

Where 0 <*c*_1_<*c*_2_< 1 are parameters that define the probability range of differences. For example, we define *PDDM*_0.95,1_ (here called PDDM95 for short) by selecting *c*_1_ = 0.95 and *c*_2_ = 1 to define the distance metric based on the right tail (5%) of the power distribution functions.

**Fig 2:**
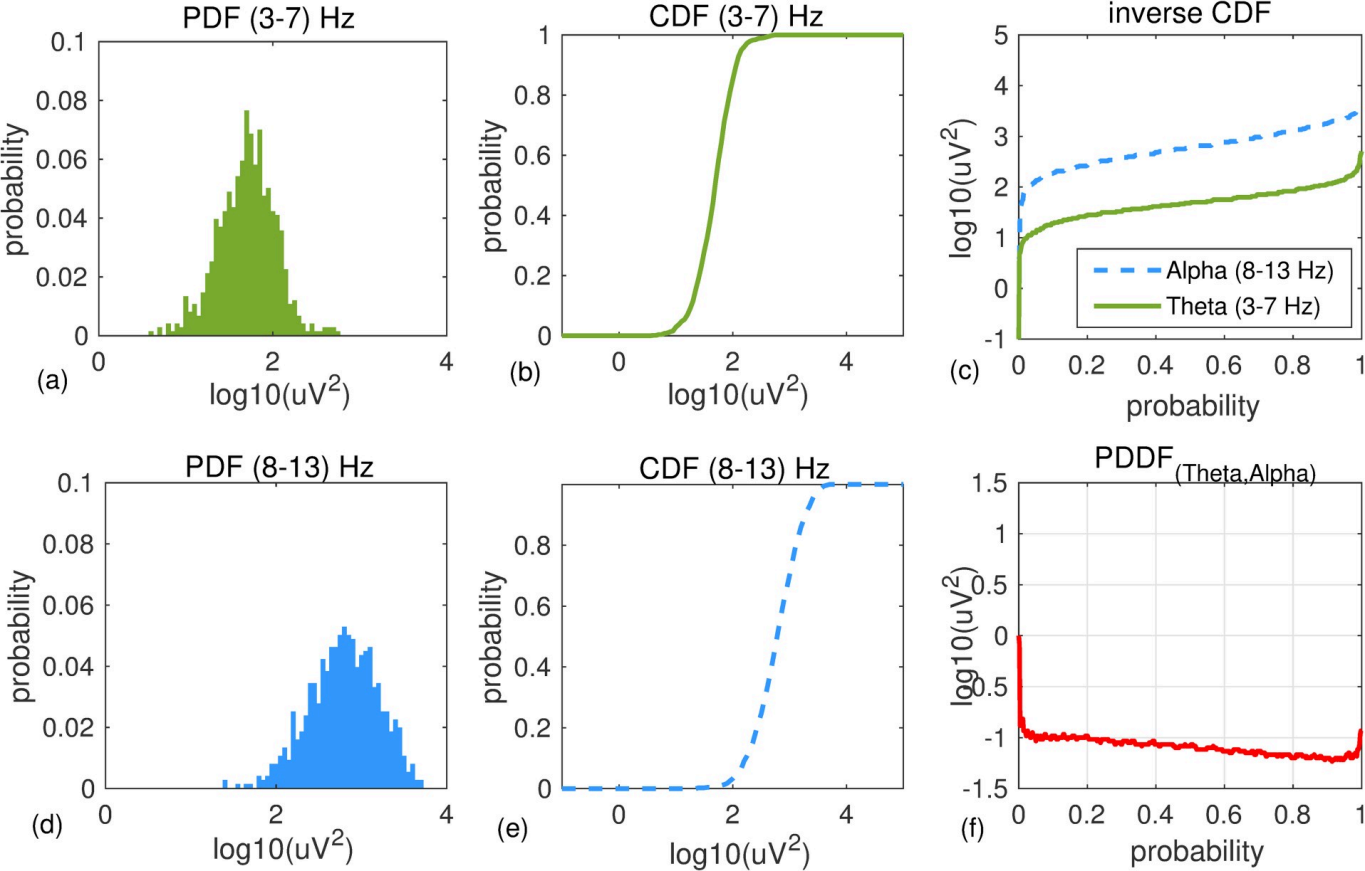
An example of PDDF for a healthy participant: An example of PDF and CDF functions for Theta (a,b) and Alpha (d,e) bandwidths plotted for EEG data recorded at channel T6 from a healthy 71 year old male participant (MMSE score = 30). Overall, the participant has higher Alpha than Theta power. Inverse CDF functions and the PDDF function are shown in (c) and (f), respectively.

**Fig 3:**
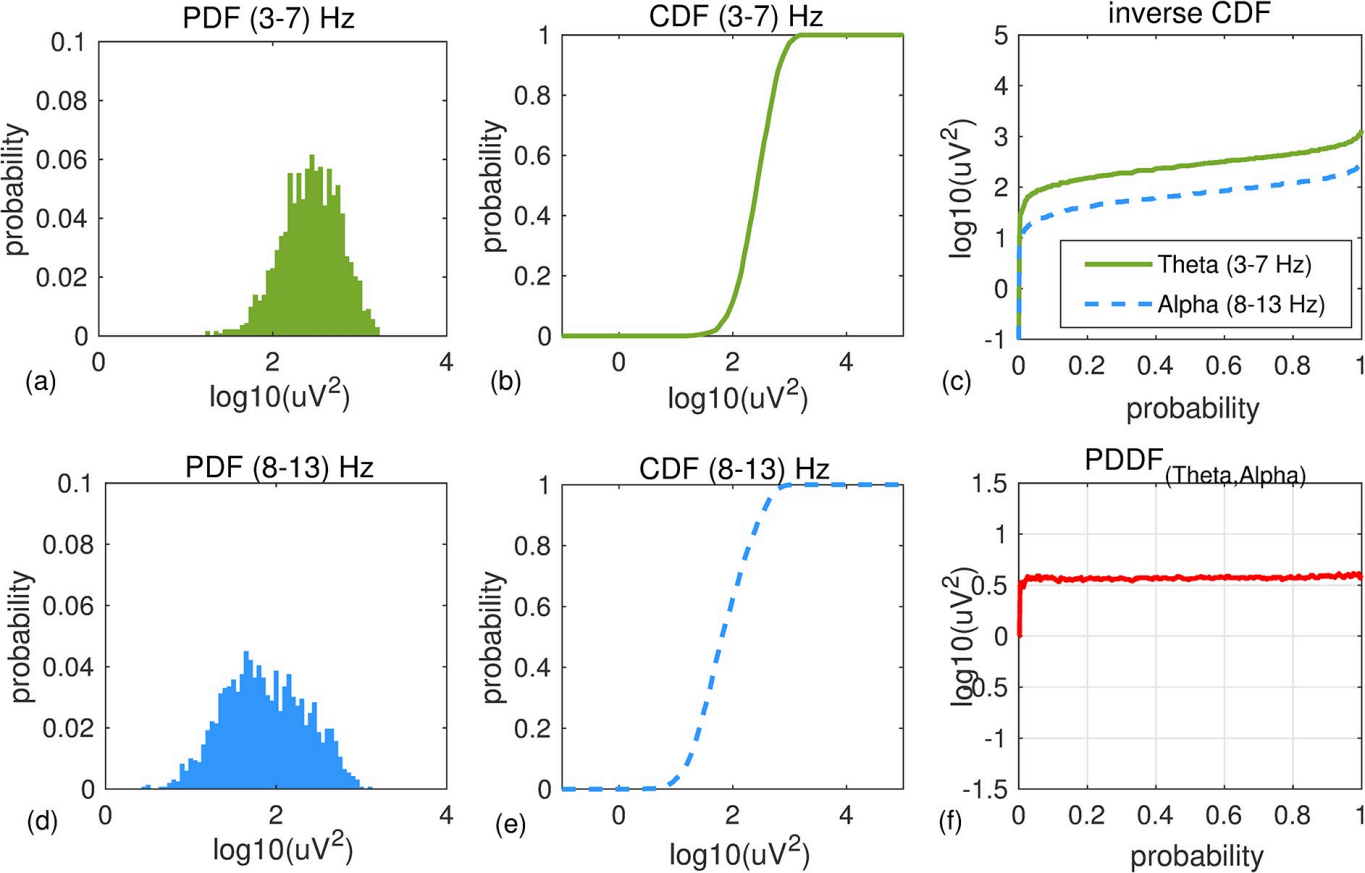
An example of PDDF for a participant with AD: An example of PDF and CDF functions for Theta (a,b) and Alpha (d,e) bandwidths plotted for EEG data recorded at channel T6 from a 74 year old female participant with AD (MMSE score = 18). Overall, the participant has higher Theta than Alpha power. Inverse CDF functions and the PDDF function are shown in (c) and (f), respectively.

**Example.** Fig 2(F) shows an example of EEG power distribution difference function (PDDF) computed for data recorded at channel T6 from a healthy 71-year-old male participant during 300 seconds of resting state eyes closed. Empirical PDF and CDF of EEG power in Theta 3–7 Hz (a,b) and Alpha 8–13 Hz (d,e) are shown. Inverse CDF functions for both bandwidths (Fig 2C) and their differences (PDDF: Fig 2D) are shown. **Fig 3** shows the same graphs for a 74-year-old female with Alzheimer’s disease.

The PDDF functions for participants in each participant group were averaged. *PDDM*_0.95,1_(*Theta*,*Alpha*) for each participant at each channel location was computed.

### Pattern classification analysis

Linear Discriminant Analysis (LDA) was used to design 2 separate binary classifiers to classify AD versus HC3 and MCI versus HC3 groups. HC3 was used as the proper age matched controls for AD and MCI participants. Input variables included all relative PSDs averaged across sessions in all frequency bins and bands recorded at all channels and regions (n = 1530). Principle component analysis (PCA) was used as an unsupervised method for feature dimensionality reduction before classification. The first *K* principle components that account for more than 98% of the cumulative proportion of explained variance were selected for downstream analyses with the remaining components rejected. The selected principle components were used as feature vectors for the supervised binary classifier.

AD (versus HC3) and MCI (versus HC3) classifiers were designed and evaluated using a bootstrapping technique with random under-sampling as described below. In each iteration of the bootstrapping, *n* subjects, where *n* is the number of participants in the minority class, were randomly selected from the majority class resulting in a classifier with a balanced data set (i.e. with equal number of subjects in each class). The classifier was trained and tested using the new balanced data set for each of the 100 iterations. In each iteration, the classifier was trained and evaluated using a leave-one-out (LOO) cross validation technique to avoid overfitting. LOO method consisted of 2*n* times training and testing, each time leaving one subject out, training on 2*n*-1 remaining subjects and testing on the one that was left out. The true positive rate (TPR), false positive rate (FPR), accuracy (percent correctly classified) and the receiver operating characteristic (ROC) curve were computed for each of the 100 bootstrapping iterations and averaged across all iterations. Performance of each of the two classifiers was assessed using overall AUC and accuracy after cross-validation and bootstrapping. The final classifier was selected by averaging the coefficients in all the 100 iterations to avoid overfitting to the set of participants used for that iteration. The posterior probabilities of MCI (using the MCI vs HC3 classifier) and AD (using the AD vs HC3 classifier) were computed for all the participants. The MCI classifier was also tested on the AD group and the AD classifier was also tested on the MCI group.

### Statistical analysis

Independent two sample t-tests with Satterthwaite approximation (Welch’s t-tests) were used to identify any significant differences between the groups (HC1, HC2, HC3, MCI, AD) at each EEG channel for each frequency bands (Delta, Theta, Alpha), and derived EEG measures (Theta-to-Alpha ratio, PDDM, and coherence). Raw effect sizes were standardized using Hedges’ g to permit comparisons between the different testing scenarios. Correlation analysis was employed to test fit of EEG measures with clinical neuropsychological tests commonly used to assess AD-associated symptomatology, including the Mini-Mental State Examination (MMSE), the Dementia Rating Scale (DRS-2). All tests were two-tailed. The α criterion for significance was set to 0.05. Results were not corrected for multiple comparison unless otherwise stated. In cases where the results were corrected for multiple comparison, FDR (False Discovery Rate) method of Benjamini-Hochberg procedure was used.

## Results

### Average power spectral density in normal aging and Alzherimer’s disease

**Fig 4:**
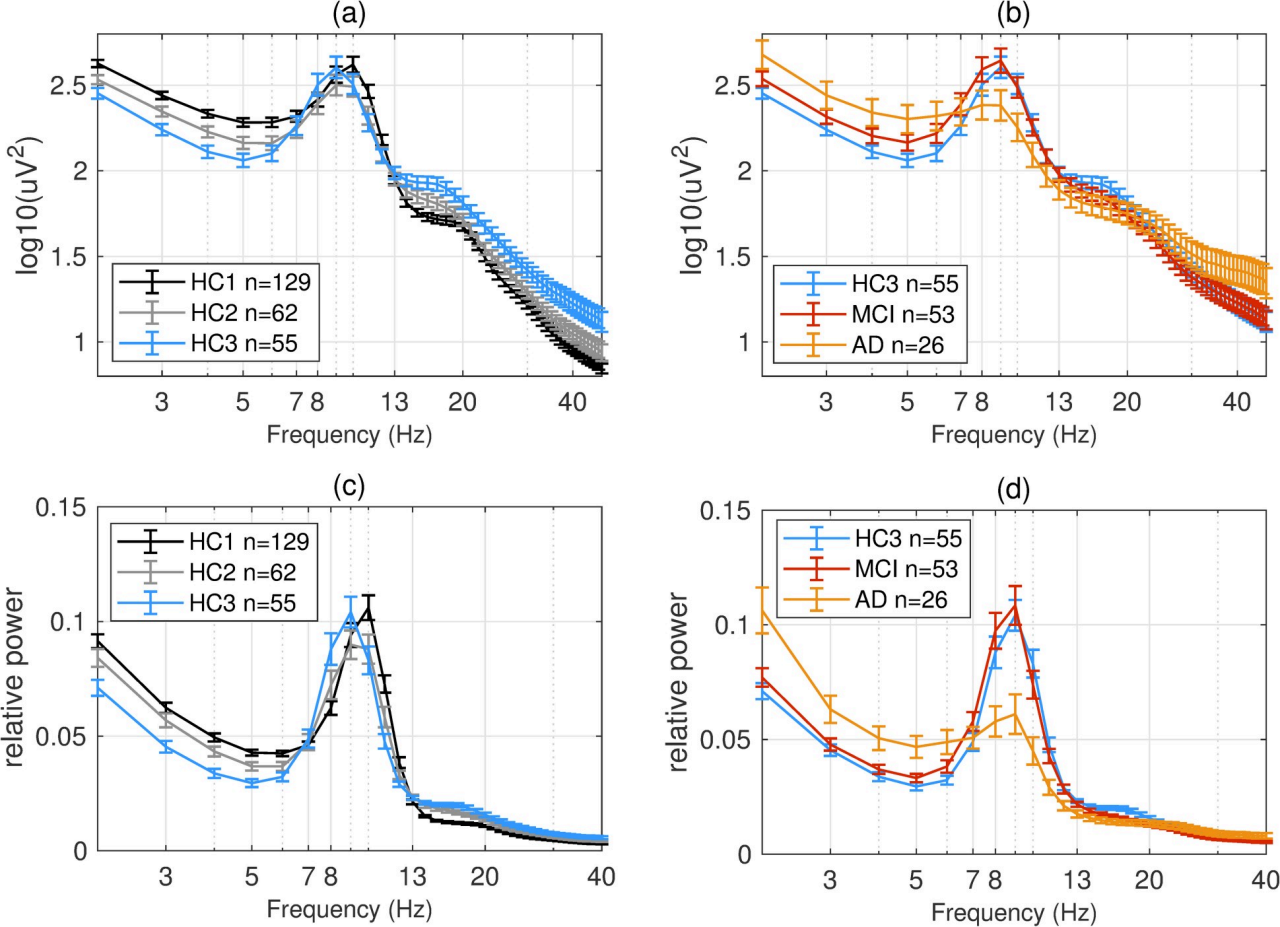
Overall group average PSDs: Group averages of global (averaged across time and channel locations) absolute (a,b) and relative (c,d) PSDs for all participant groups. In healthy participants (a,c) older participant groups show lower power at low frequencies (1–7 Hz). In contrast, MCI and AD groups (b,d) show increased power in low frequencies compared to age matched controls (HC3). At high frequencies (>20 Hz) AD participants show increased absolute power in the same direction as normal aging albeit with larger effect size.

**Fig 5:**
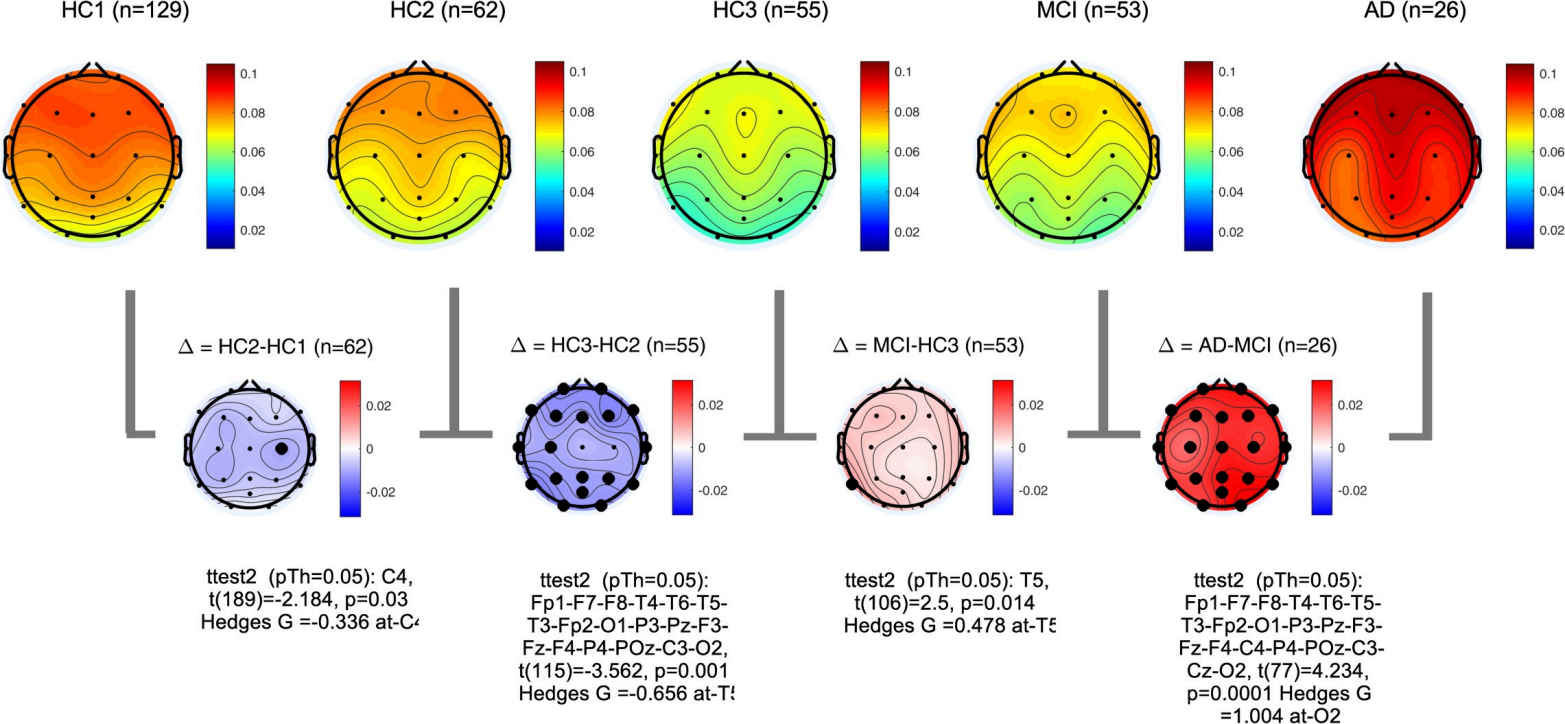
Topographical maps of Delta power: Group average topographical maps of Delta power (1–3 Hz) for all participant groups (top) and average group difference (bottom). Channels with significant differences (p<0.05) are marked with black circles.

**Fig 6:**
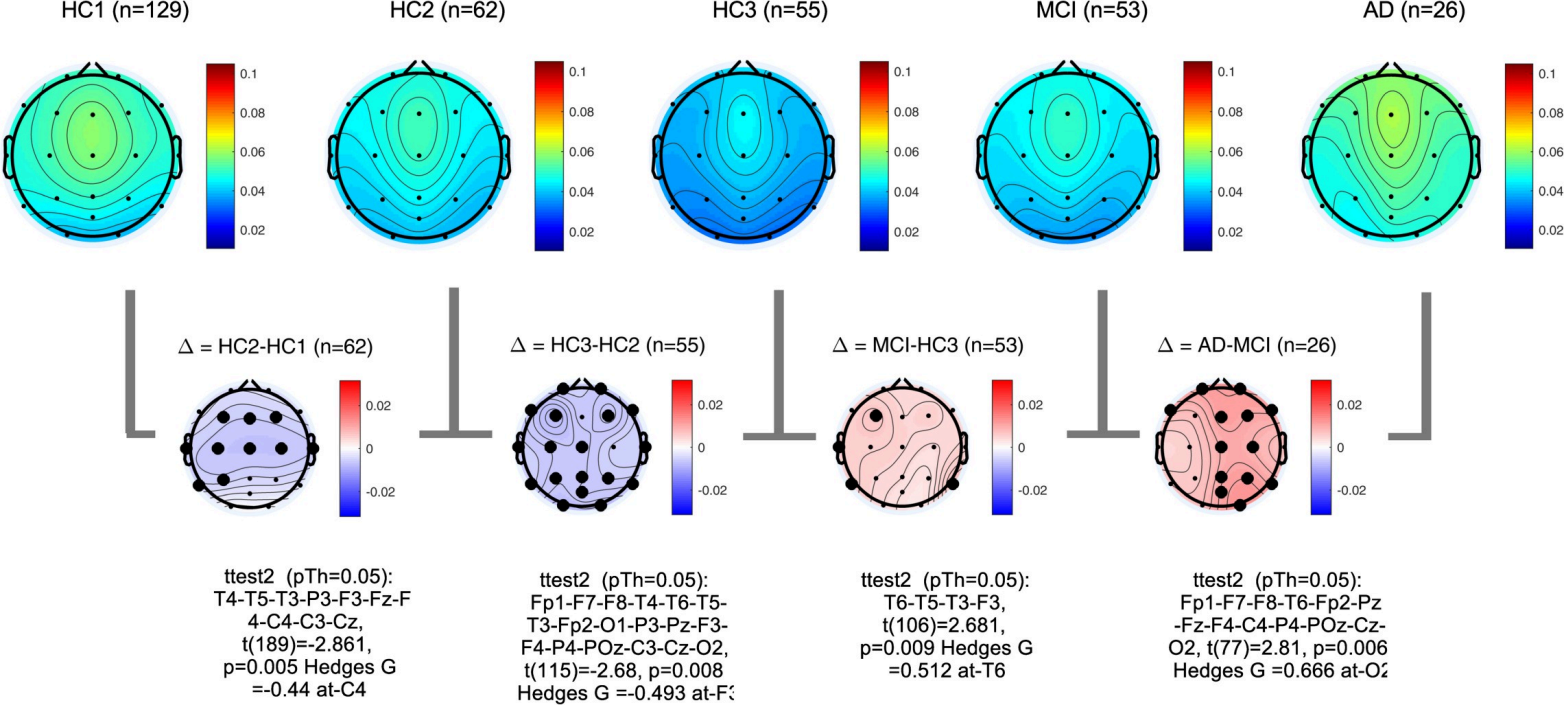
Topographical maps of Theta power: Group average topographical maps of Theta power (3–7 Hz) for all participant groups (top) and average group difference (bottom). Channels with significant differences (p<0.05) are marked with black circles.

**Fig 7:**
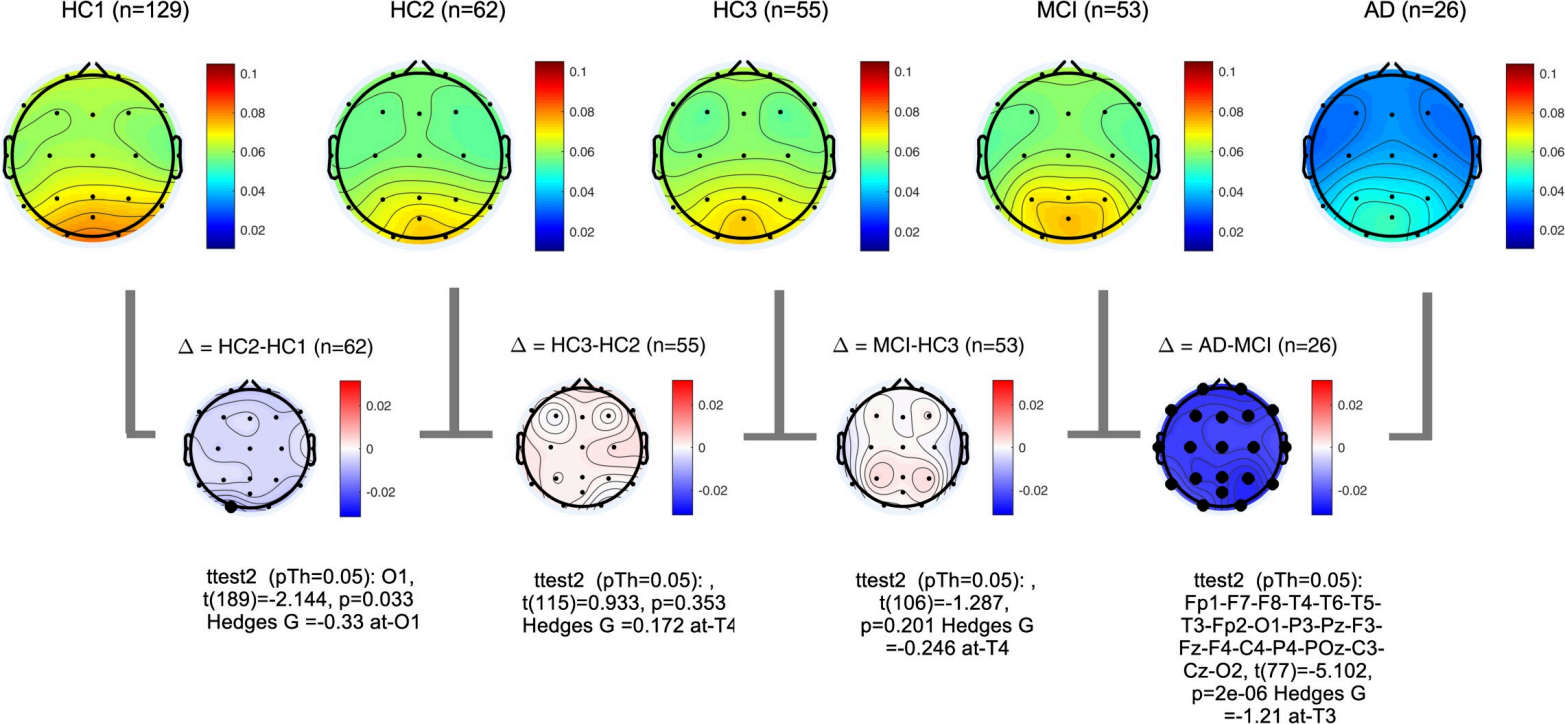
Topographical maps of Alpha power: Group average topographical maps of Alpha power (8–13 Hz) for all participant groups (top) and average group difference (bottom). Channels with significant differences (p<0.05) are marked with black circles.

**Fig 8:**
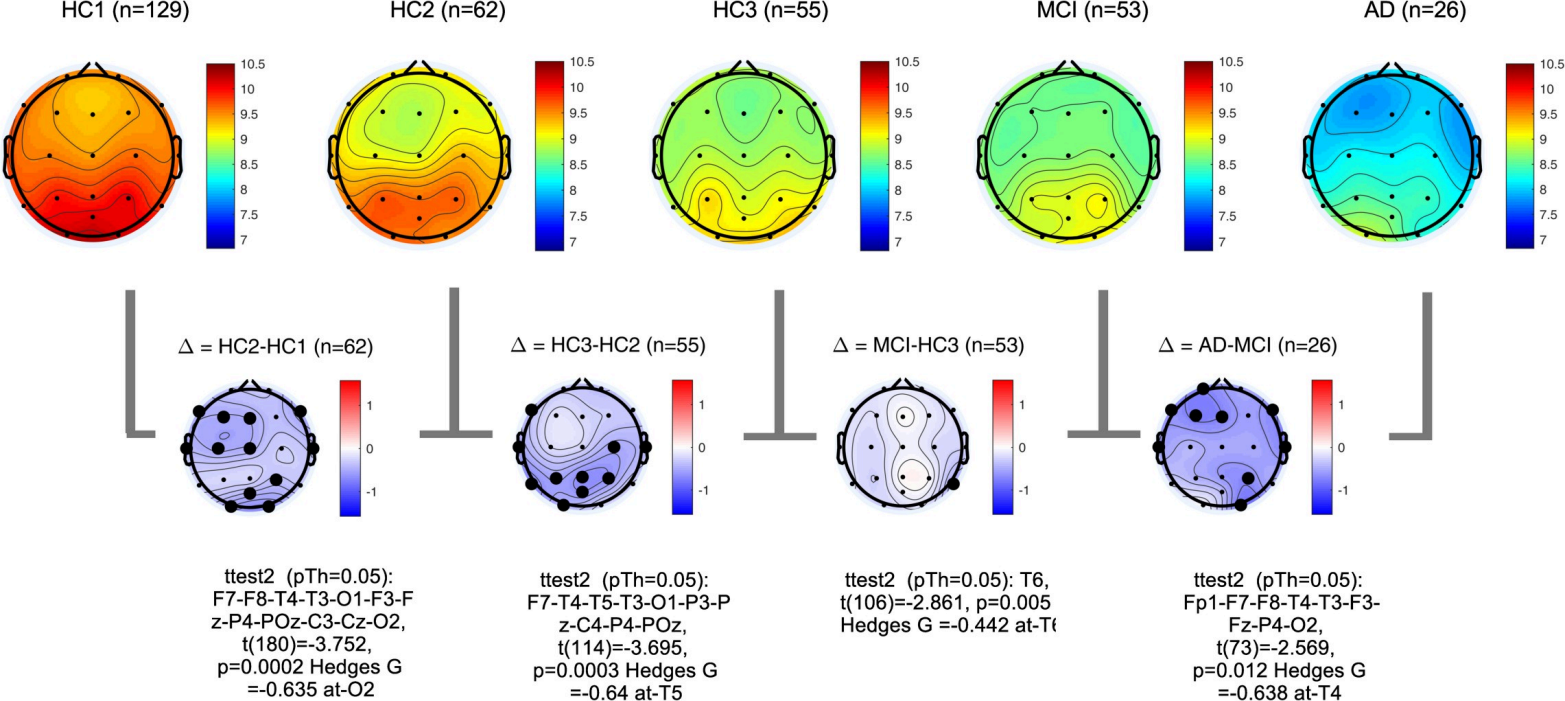
Topographical maps of Alpha peak frequency: Group average topographical maps of Alpha peak frequency for all participant groups (top) and average group difference (bottom). Channels with significant differences (p<0.05) are marked with black circles.

**Fig 9:**
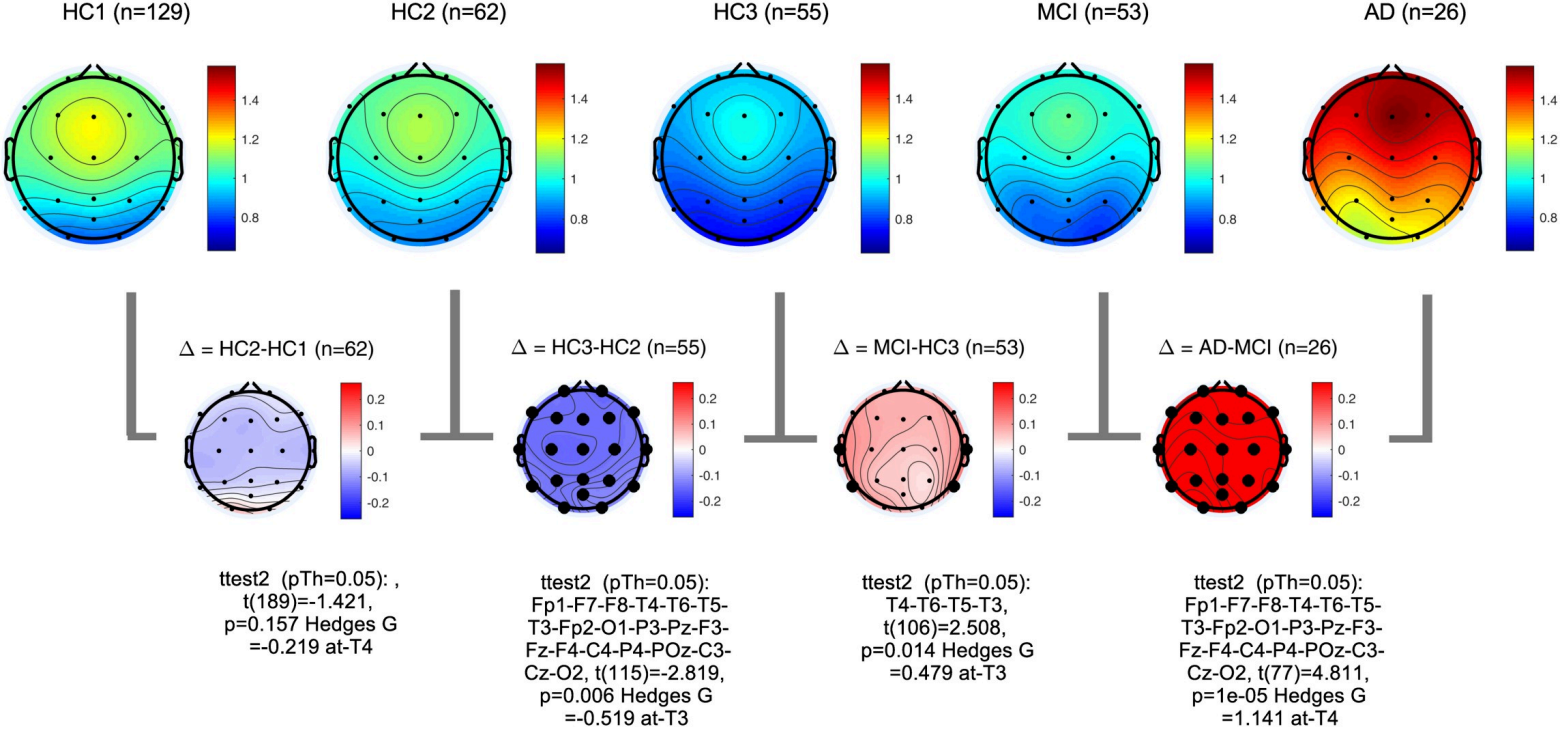
Topographical maps of Theta to Alpha ratio (TAR): Group average topographical maps of TAR for all participant groups (top) and average group differences (bottom). Channels with significant differences (p<0.05) are marked with black circles.

**Fig 10:**
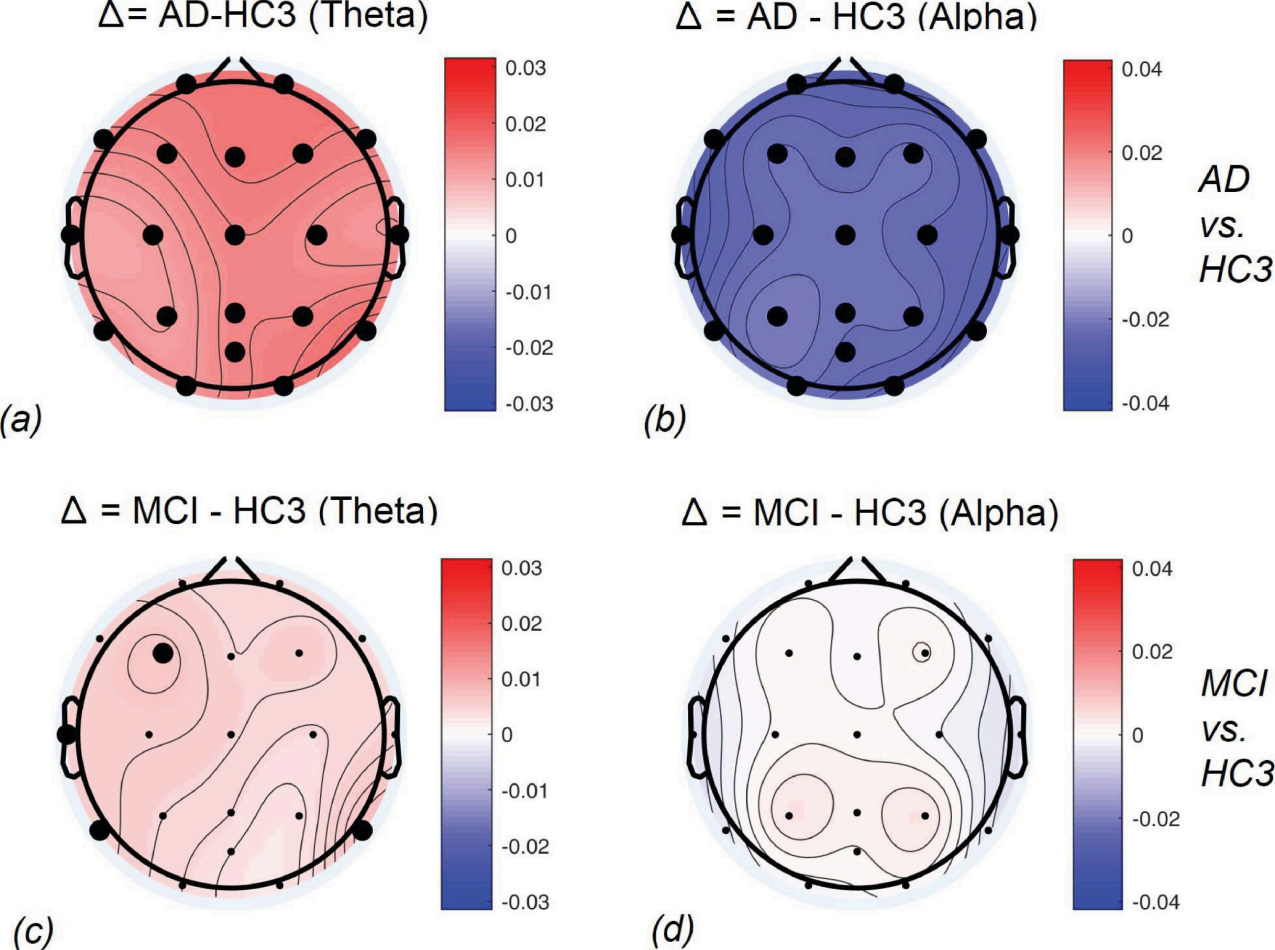
Topographical maps of the AD and MCI groups compared to controls: Differences between AD and HC3 (a,b) and MCI and HC3 (c,d) in Theta and Alpha bands. significant differences are marked with black circles.

PSDs during resting state eyes closed for each participant at each EEG channel was computed according to the methods section. The global PSD power (average power across all recording channels) was also computed for each participant. Group averages of global PSDs versus frequency are plotted in **Fig 4** for both absolute (a,b) and relative power (c,d). Overall and consistent with the existing literature, in healthy controls, average power at low frequencies (Delta and Theta range, 1–7 Hz) are decreasing with age (**Fig 4A** and **4**C) [51–53]. In contrast to this healthy aging effect, the AD group showed increased power in slow frequencies (**Fig 4B** and **4**D) compared to age matched controls (HC3). The MCI group showed the same trend albeit smaller in size. The group averages for all frequency bands in each participant group are also listed in the supplementary section. These results are overall trends and do not account for regional changes in power measured at different channels. To better understand regional changes, topographical maps of power spectral densities for each participant group at predefined standard frequency bands as well as the ratio of power in Theta to Alpha bands were plotted in **Figs 5**–**9**. In each figure, channels with significant differences between groups were marked on the figure and the channel with the smallest p-value (two sample t-test) as well as the normalized effect size (Hedges G) and t-statistics were reported in **Table 4**. Topographical maps of the differences between MCI, AD and age matched HC3 group have been plotted in **Fig 10** for Theta and Alpha band separately.

**Table 4:** The channel with the most significant differences between participant groups, the corresponding normalized effect sizes hedges-G, t-statistics, and p value.

G(channel) t(df), p	Δ = HC2-HC1	Δ = HC3-HC2	Δ = MCI-HC3	Δ = AD-MCI	Δ = AD-HC3
Rel. Delta 1–3 Hz	G(C4) = - 0.34	G(T5) = - 0.66	G(T5) = 0.48	G(O2) = 1.0	G(T5) = 1.28
	t(189) = -2.1	t(115) = -3.56	t(106) = 2.5	t(77) = 4.23	t(79) = 5.44
	p = 0.03	p = 10^−3^	p = 0.01	p = 10^−4^	p = 5x10^-7^
Rel. Theta 3–7 Hz	G(C4) = -0.44	G(F3) = -0.49	G(T6) = 0.51	G(O2) = 0.67	G(T6) = 1.02
	t(189) = -2.86	t(115) = -2.68	t(106) = 2.68	t(77) = 2.81	t(79) = 4.34
	p = 5x10^-3^	p = 8x10^-3^	p = 9x10^-3^	p = 6x10^-3^	p = 4x10^-5^
Rel. Alpha 8–13 Hz	G(O1) = -0.33	Not Significant	Not Significant	G(T3) = -1.21	G(T3) = -1.45
	t(189) = -2.14			t(77) = - 5.1	t(77) = -6.14
	p = 0.03			p = 2x10^-6^	p = 3x10^-8^
Alpha Peak Frequency	G(O2) = -0.36	G(P4) = -0.66	Not Significant	G(T4) = -0.64	G(T6) = -0.9
	t(189) = -2.32	t(115) = -3.6		t(73) = -2.6	t(75) = -4.03
	p = 0.02	p = 5x10^-4^		p = 0.01	p = 0.001
${TAR}_{\frac{3-7\;Hz}{8-13\;Hz}}$	Not Significant	G(T3) = -0.52	G(T3) = 0.48	G(T4) = 1.41	G(T4) = 1.59
		t(115) = -2.82	t(106) = 2.5	t(77) = 4.81	t(77) = 6.77
		p = 6x10^-3^	p = 0.01	p = 10^−5^	p = 2x10^-9^
*PDDM*_0.95_ (*Theta*,*Alpha*)	G(O1) = 0.34	G(T5) = -0.48	G(T3) = 0.79	G(T4) = 1.04	G(T3) = 1.78
	t(189) = 2.2	t(115) = -2.6	t(106) = 4.1	t(77) = 4.38	t(79) = 7.56
	p = 0.03	p = 0.01	p = 10^−4^	p = 4x10^-5^	p = 6x10^-11^

In alignment with prior published reports, participants with AD clearly evidenced significant increases in the slower frequencies (Delta, Theta) and decreases in the faster frequencies (Alpha, Beta) in comparison to MCI and healthy controls. It is likely that this combination of changes in slow and fast frequencies represents an important EEG biomarker of progression to AD. The frequency of the Alpha peak in the AD group was significantly smaller than both MCI and HC3 groups (**Table 4**, **Fig 8**, and **S2 Fig** in the supplementary section). It is worth noting that measured alpha peak frequencies are affected by the frequency resolution of our methods (1Hz).

The MCI group on average showed a moderate increase in both Delta, Theta and Theta-to-Alpha ratio which was significant mainly at temporal channels. The MCI group did not show a significant decrease in Alpha power (**Fig 7**). The AD group, on average, showed significantly higher Delta, Theta and Theta-to-Alpha power ratio and significantly lower Alpha power.

### PSD distribution analysis

**Fig 11:**
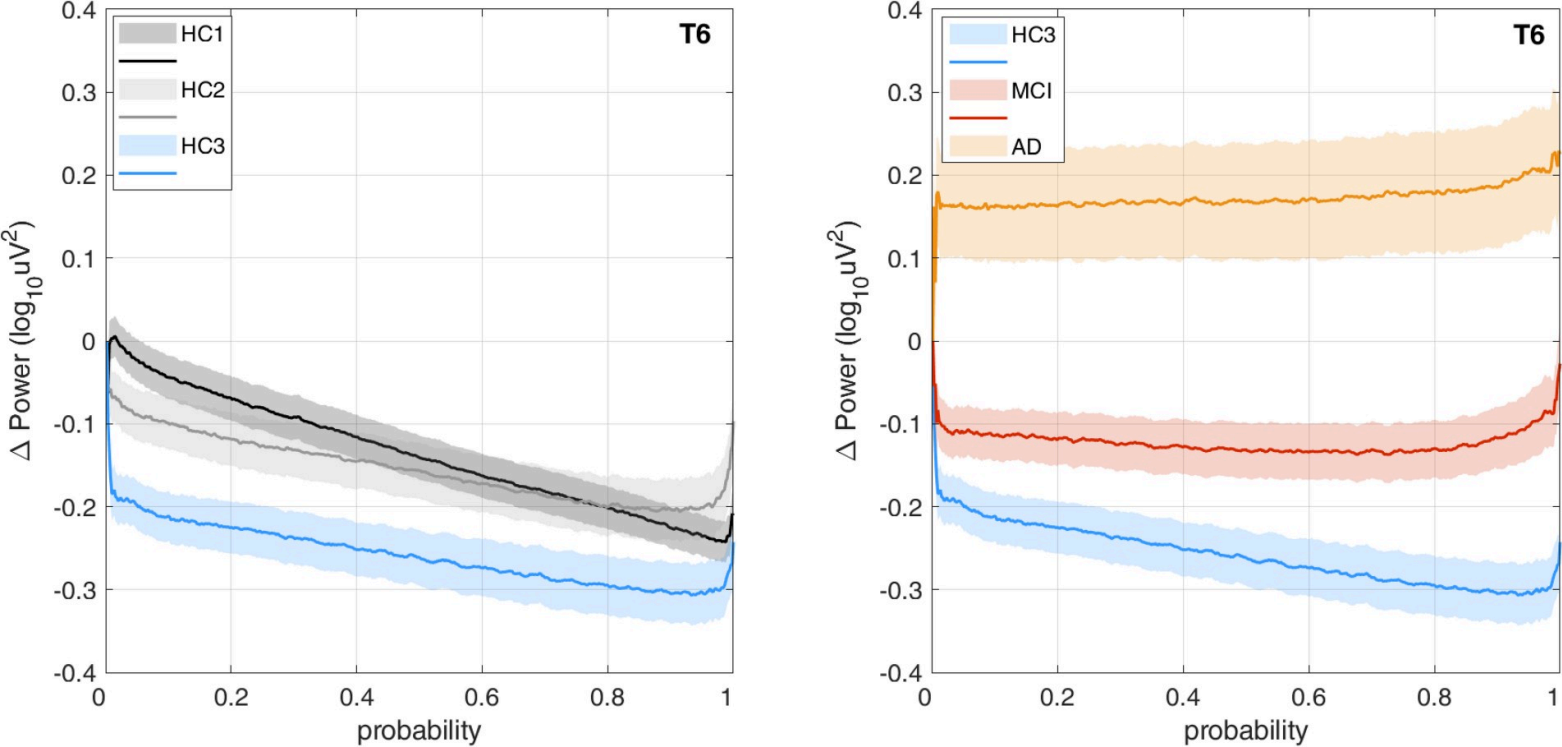
PDDF_(Theta,Alpha)_ power distribution difference functions: Graphs of group average PDDF functions demonstrating the difference between Theta and Alpha band power distributions at channel T6 plotted for each participant group. Solid lines represent group means and shaded areas show standard error of the means.

**Fig 12:**
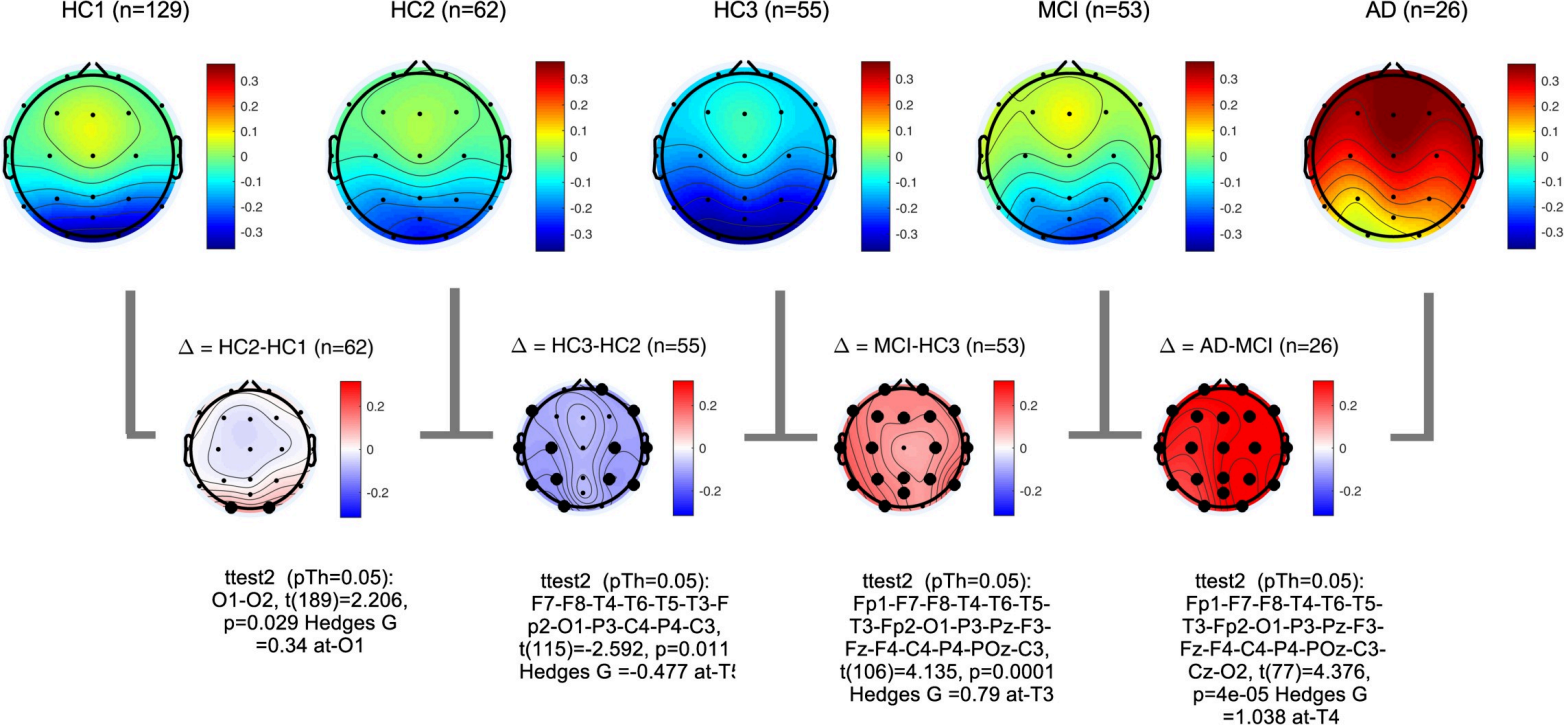
Topographical maps of *PDDM*_0.95,1_(*Theta*,*Alpha*): Group average topographical maps of PDDM for all participant groups (top) and average group differences (bottom). Channels with significant differences (p<0.05) are marked with black circles.

**Fig 13:**
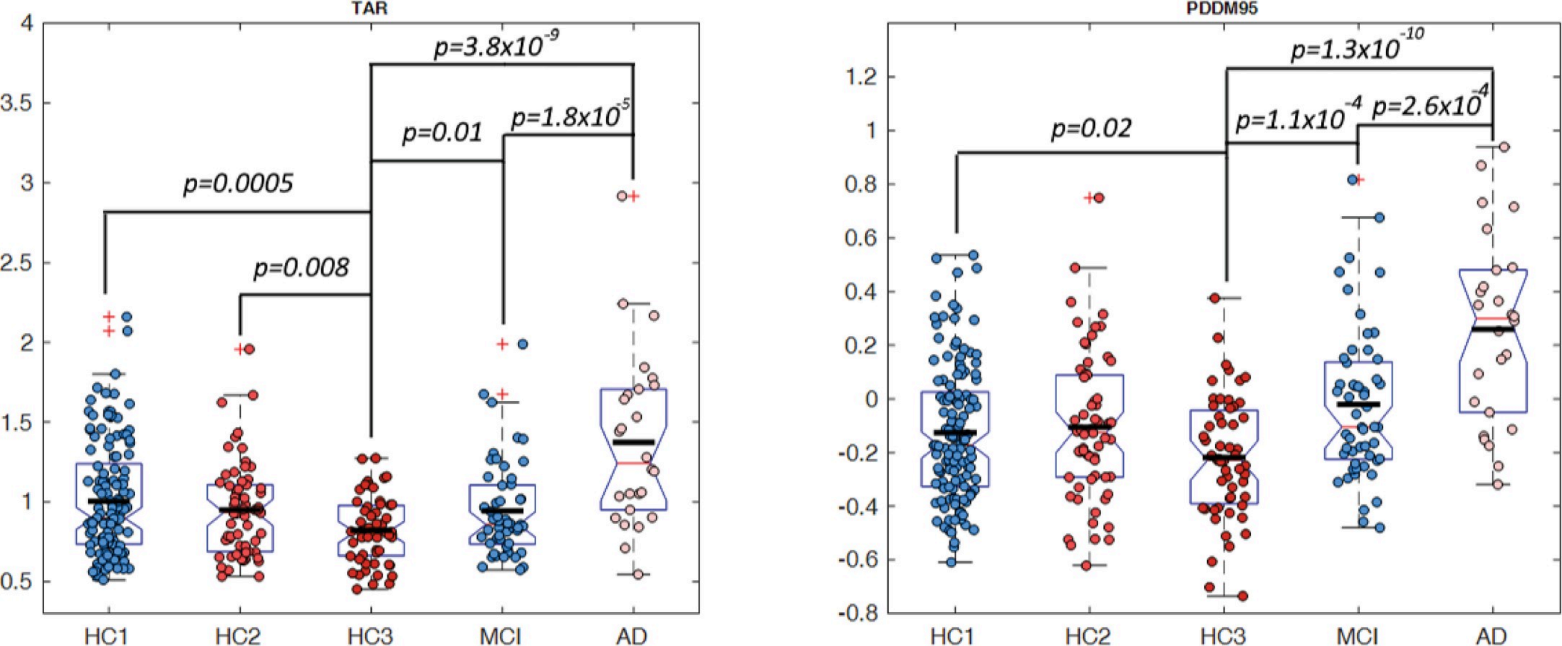
Group average TAR and PDDM95 at temporal areas: Group average of TAR (left) and PDDM (right) at temporal areas. Significant differences between groups were marked with the p-value of a two-sample t-test.

The difference between distribution of power at Theta (3–7 Hz) and Alpha (8–13 Hz) bands was computed according to Eq 1 in the methods section. **Fig 11** shows the proposed difference function *PDDF*_(*Theta*,*Alpha*)_(*u*) averaged across all participant groups plotted for channel T6 as an example. The graph illustrates the finding that, on average, healthy controls have higher Alpha power than Theta power resulting in a PDDF function with negative values (situated under the zero baseline) and a negative slope. The MCI group, on average has less negative PDDF values with smaller slope. The AD group shows a reverse trend with higher Theta power than Alpha (average PDDF is above the zero baseline). The plots demonstrate that the difference between MCI and HC3 is greater at the higher probabilities (shown on the x-axis) supporting our hypothesis that in the MCI group, higher Theta to Alpha ratios may be seen not throughout the session but starting to emerge in selected epochs. Therefore, the interval between 0.95 and 1 was selected in an ad hoc manner to compute PDDM according to Eq 2. **Fig 12** shows the topographical map of *PDDM*_(0.95,1)_(*Theta*,*Alpha*) for each participant group. PDDM is significantly higher in the MCI group compared to the age matched HC3, in all channels except Cz and O2. The channel with the smallest p-value between participant groups are similarly reported in **Table 4**. The results show that PDDM provides better discrimination between MCI and HC3 group (compared to TAR) in that, the effect sizes are larger (G = 0.79 at T3), p-values are smaller (t(106) = 4.1, p = 10^−4^) and more widespread across the scalp areas (18 out of 20 channels with p<0.05). However, comparing the AD and MCI groups, PDDM and TAR are comparable in terms of effect size and significance. This will support the initial research hypothesis that using PDDM may provide more sensitive measure of cognitive decline in earlier stages of cognitive decline in MCI where EEG changes may not be otherwise significant. The results show that most significant differences between the MCI/AD groups and the age matched controls (HC3) are in temporal channels (T3,T4,T5,T6). We computed the average EEG measures at temporal sites by averaging each EEG measure across the four temporal channels (T3,T4,T5,T6) and compared the group means. **Fig 13** shows group means for both TAR and PDDM at temporal sites. The group means of temporal TAR for the HC1,HC2,HC3, MCI, and AD groups are 1.00, 0.95,0.82,0.94, and 1.37, respectively. These group means for PDDM95 are -0.13,-0.10,-0.22,-0.02, and 0.26, respectively. We define MCI effect size as the difference between the MCI group mean and the HC3 group mean and AD effect size as the difference between the AD group mean and the HC3 group mean. Consequently, the results show that using TAR, MCI effect size is 22% of the AD effect size while using PDDM95, MCI effect size is 41% of the AD effect size. Furthermore, the difference between group means of the HC1 and HC3 (aging effect) is 33% of the AD effect size using TAR and 19% of the AD effect size using PDDM95. These results suggest that PDDM95 is more sensitive to cognitive decline and less sensitive to normal aging.

### EEG coherence

**Fig 14:**
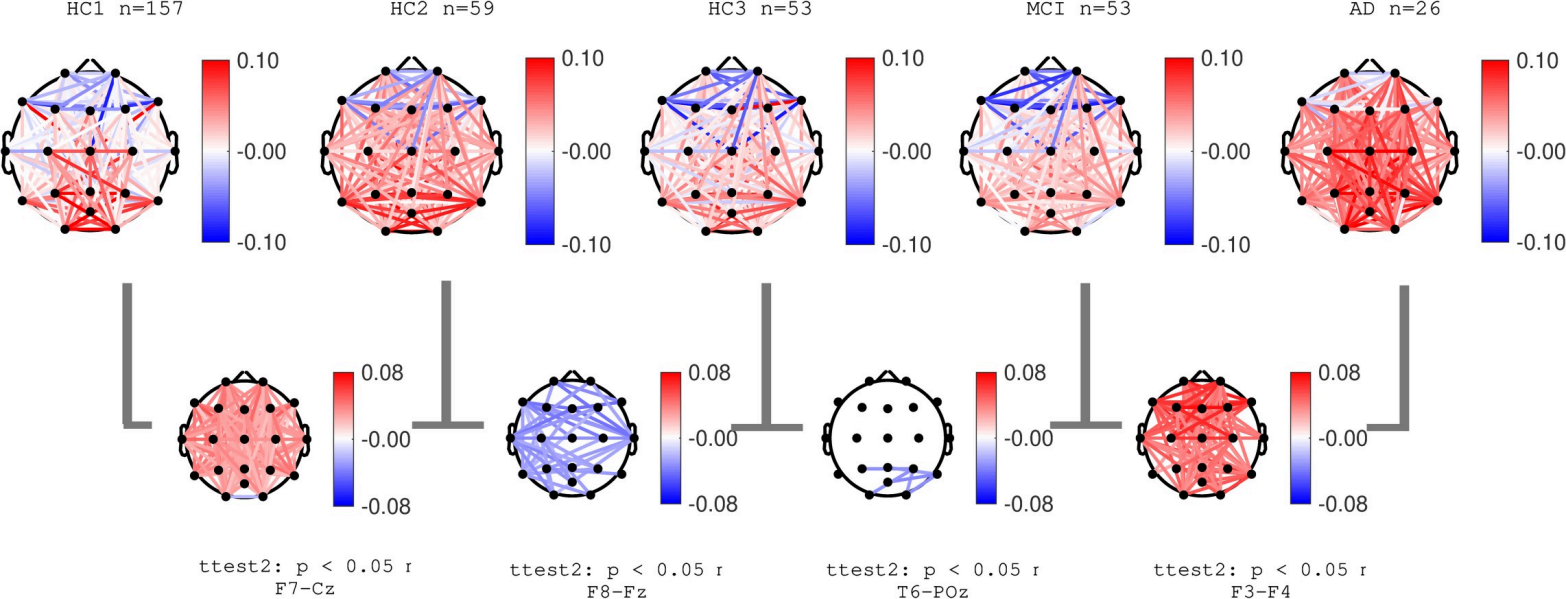
Topographical maps of Delta coherence: Topographical maps of normalized coherence in Delta band across pairs of channels in each participant groups. Red (and blue) color represent higher (and lower) coherence compared to average across all frequencies.

**Fig 15:**
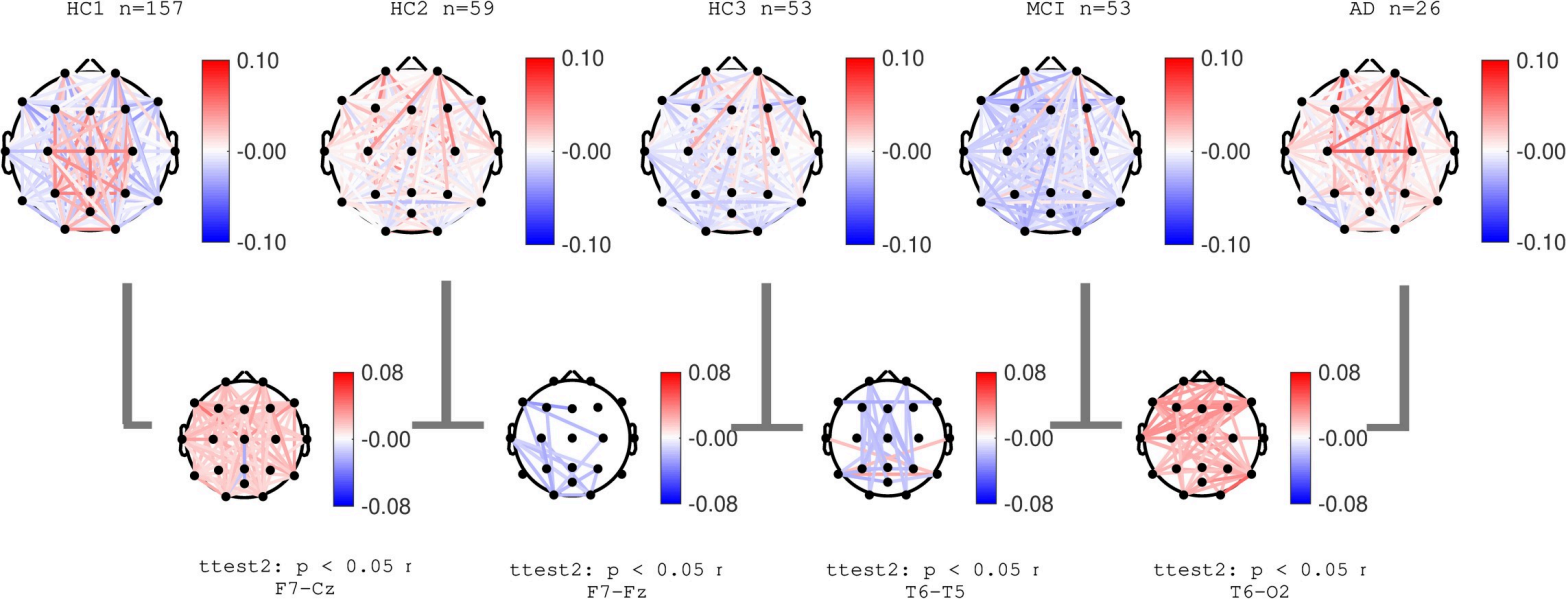
Topographical maps of Theta coherence: Topographical maps of normalized coherence in Theta band across pairs of channels in each participant groups. Red (and blue) color represent higher (and lower) coherence compared to average across all frequencies.

**Fig 16:**
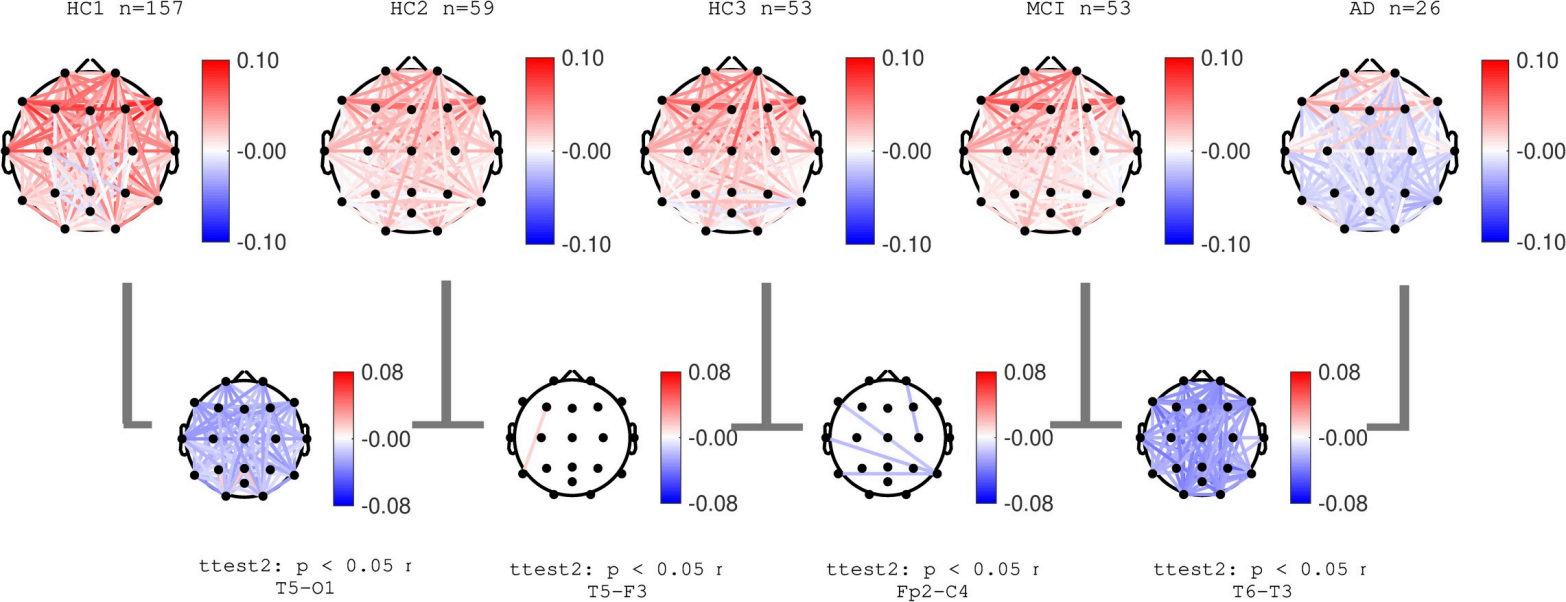
Topographical maps of Alpha coherence: Topographical maps of normalized coherence in Alpha band across pairs of channels in each participant groups. Red (and blue) color represent higher (and lower) coherence compared to average across all frequencies.

Normalized coherence for all pairs of channels was computed as described in the methods section. **Figs 14**–**16** show group average topographical maps of normalized coherences between pairs of channels for Delta, Theta and Alpha bands, respectively. The plots are color coded to demonstrate the normalize coherence levels at each frequency bands. Red colors show coherences greater than average and blue colors show less than average coherence. The pairs of channels with significant difference between groups are shown and the pair of channels with smallest p-value is named under each plot. The results show that normal aging effect consists of increased normalized coherence in Delta and Theta bands after 40 years age (HC2) followed by a decrease after 60 years of age (HC3) as well as decrease in alpha coherence after 40 years of age (HC2). The AD group showed a clear significant increase in normalized Delta and Theta coherence and significant decrease in Alpha coherence consistent with PSD results. These changes in the MCI group (compared to HC3) were only partially observed in limited pairs of channels. These data are also in agreement with prior published reports, the majority reporting decreased in Alpha coherence associated with the progression of dementia [13,23,43,49,61]. More recent reports also identified an increase in coherence in the Theta band associated with AD severity [62,63].

### Summary of the EEG measures

Table 5 shows a summarized descriptive overview of the findings. The results show that overall, PSD based changes in our proposed EEG measures in normal aging are monotonic with significant changes after 40 or 60 years of age whereas coherence-based measures may show conflicting trends based on the age group. In the AD group, the measures are generally robust with widespread and significant changes that oppose the trend of normal aging except for Alpha power and Alpha power frequency where normal aging and AD effects are aligned with each other.

**Table 5:** Overall descriptive effects of AD and MCI on EEG measures compared to normal aging.

EEG Measure	Normal Aging	AD	MCI
Relative Delta Power	↓↓ Decreasing (mainly significant after 60 years)	↑↑ Widespread significant increase	↑ Increased, significant only at left temporal area.
Relative Theta Power	↓↓ Significant decrease at most channels	↑ Increased power mainly significant across right hemisphere channels.	↑ Increased, and significant only in selected channels T3,T5,T6, F3
Relative Alpha Power	**~~** No significant change after 40 years old	↓↓ Significant and widespread decrease	**~~** Not significantly different than controls.
Alpha Peak Frequency	↓ Decreasing with age, only significant at limited areas (occipital and temporal)	↓↓ Significant and widespread decrease	**~~** Not significantly different than controls (except one channel only)
Theta-to-Alpha Ratio (TAR)	↓↓ Decreasing with age (widespread and significant after 60 years old)	↑↑ Significant and widespread	↑ Increased and significant only in temporal areas
*PDDM*_(0.95,1)_(Theta,Alpha)	↓ Decreasing with age after 60 years (significant for most channels)	↑↑ Significant and widespread increase	↑↑ Significant increase at most channels.
Norm. Delta Coherence	↑*↓ Significant increase after 40 years old followed by significant decrease after 60 years old	↑↑ Significant and widespread increase.	↓ Limited localized decrease in the right temporal and occipital areas
Norm. Theta Coherence	↑*↓ Significant widespread increase beyond 40 years old followed by significant localized decrease beyond 60 years old	↑↑ Significant and widespread increase	Significant decrease mainly across anterior-posterior areas with significant increase across left to right temporal areas
Norm. Alpha Coherence	↓↓ Significant and widespread decrease after 40 years old and almost no change after 60 years old	↓↓ Significant and widespread decrease	Significant decrease only in limited areas from right temporal to left-temporal and left-frontal

↓↓ widespread significant decrease, ↑↑ widespread significant increase, ↑*↓ not a consistent trend, ~~ no significant change, (↓**)** limited (localized) significant decrease, (↓**)** limited (localized) significant increase.

For both the AD and MCI groups, the largest effects (compared to the HC3 age matched controls) were observed using PDDM95 (**Table 4**: normalized effect sizes 1.78 and 0.79 for AD-HC3 and MCI-HC3, respectively, both measured at channel T3). All the differences between the AD and HC3 groups remained significant after FDR correction for multiple comparison among the 20 channels. However, the significant differences between MCI and HC3 did not reach the significance level after the correction, except for PDDM95 where all the channels remained significant after the correction.

### Pattern classifiers

**Fig 17:**
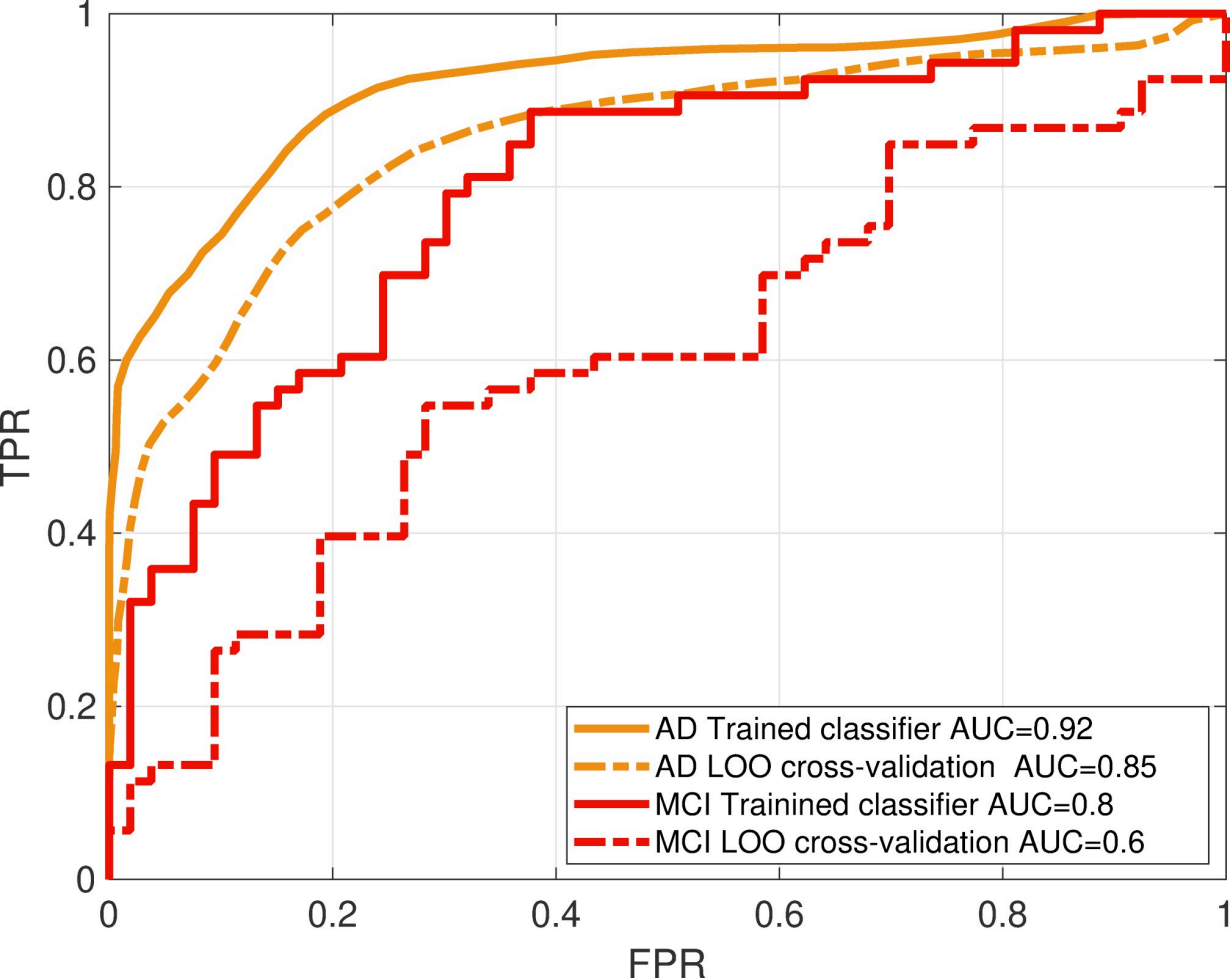
Classifiers performance: ROC curves (plots of true positive versus false positive rates) for the AD and MCI classifiers show the performance of the classification when the classifier is tested on the training data (solid line) and tested after LOO cross validation (dotted lines). The area under the curve (AUC) for each classifier shows the performance compared to chancel level (AUC = 0.5).

The MCI and AD linear classifiers were trained and validated as per the methods section. HC3 was used as the age matched control group. Principle component analysis (PCA) was used for feature reduction. As expected, and due to high correlation between PSD values at different frequencies and channel locations, PCA resulted in a highly reduced number of features (17 features for the MCI classifier and between 6 to 7 features for the AD classifier). The performance of each classifier was evaluated using the area under ROC curve. **Fig 17** shows the average ROC curve of 100 iterations, each obtained using a leave-one-out cross validation method. The average AUC for the AD and MCI classifiers were 0.85 and 0.6, respectively. **Fig 17** also shows the ROC curves without cross validation tested on the training data and hence demonstrating the level of overfitting (AUC = 0.92 and AUC = 0.8 for the AD and MCI classifiers respectively).

**Fig 18:**
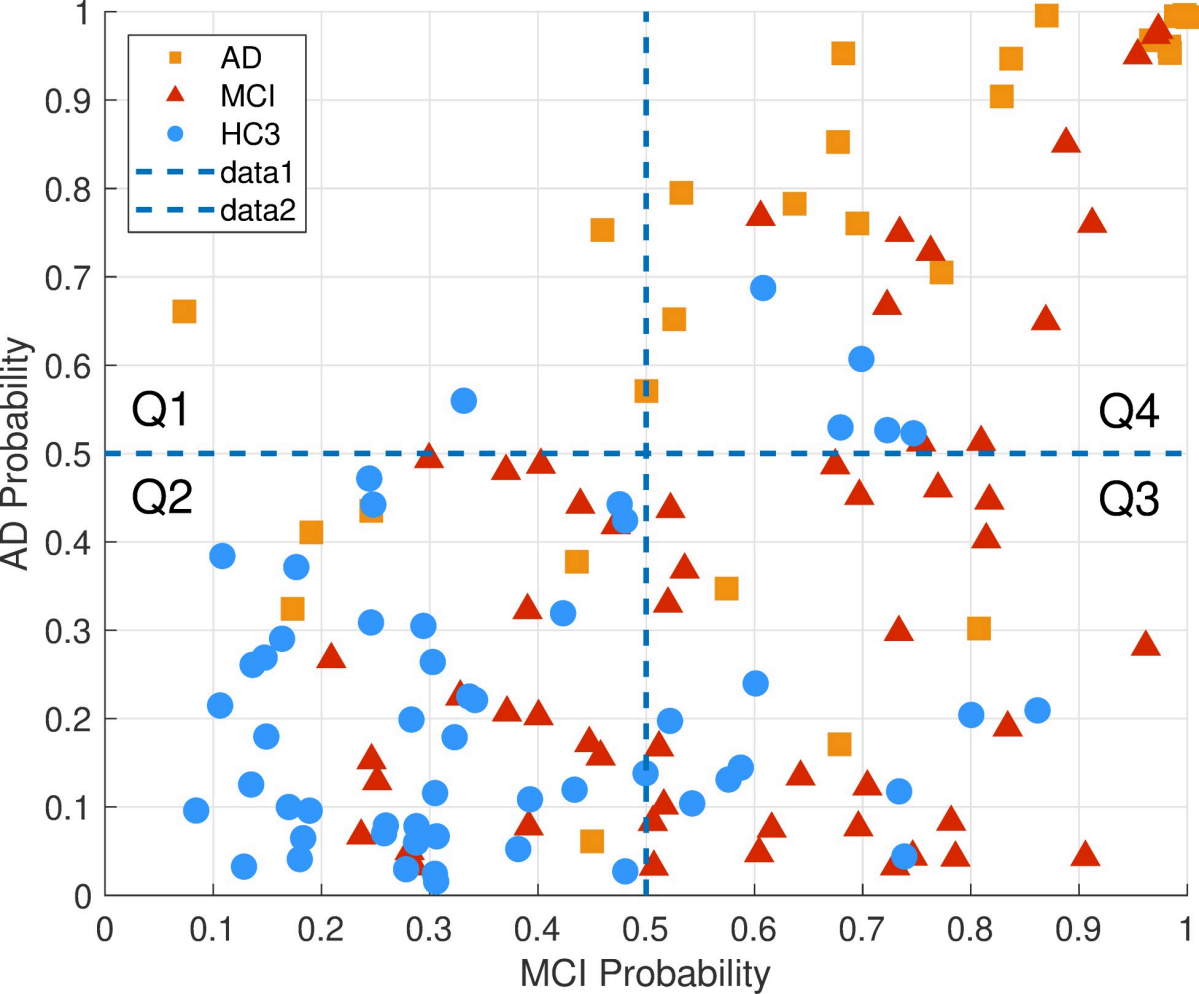
Classifiers between-group cross validation: The AD and MCI classifiers were tested on both the MCI and AD groups as well as the age matched HC3. AD probability and MCI probability indicate the posterior probabilities of AD and MCI classifiers, respectively. The probability space is divided into 4 quartiles where Q2 is the quartile with little or no cognitive decline, Q3 is the quartile with possible mild cognitive decline and Q4 is the quartile with highest cognitive decline. The hypothetical trajectory of cognitive decline in this probability space is likely passing through Q2, Q3 and Q4.

The scatter plot of the posterior probabilities in both the MCI and AD classifier is shown in **Fig 18**, demonstrating a probability space divided into four quartiles. Overall, HC3 participants have low probability in both the MCI and AD classifiers (second quartile). Conversely, the AD participants as a group have high probability of MCI and AD (fourth quartile). The MCI group are spread out across the probability space in quartiles two to four.

### Correlations between EEG biomarkers and clinical and neuropsychological scores

The correlation between EEG measures and neuropsychological scores in the AD and MCI groups are presented in this section. MMSE and Mattis DRS-2 scores for each domain were selected and compared against both TAR and PDDM95 averaged at temporal sites (Table 6). Temporal channels were selected based on the results of significant differences between the MCI,AD groups and healthy controls (HC3). Correlations at all channels were computed separately and topographical maps of the correlation coefficients were plotted in the supplementary section (**S1 Fig**).

**Table 6:** Pearson correlation between EEG measures and Neuropsychological tests of MMSE and DRS in the AD group, p-value (p) and 95% confidence intervals (CI) are reported for each correlation.

	AD probability	TAR at Temporal Sites	PDDM95 at Temporal Sites
MMSE	r(24) = -0.44, p = 0.02 CI = [-0.71, -0.07]	r(24) = -0.69, p = 10^−4^ CI = [-0.85, -0.41]	r(24) = -0.77, p = 5x10^-6^ CI = [-0.89, -0.54]
DRS-Initiation Preservation (IP)	r(18) = -0.56, p = 0.01 CI = [-0.80, -0.15]	r(18) = -0.53, p = 0.015 CI = [-0.79, -0.12]	r(18) = -0.57, p = 8x10^-3^, CI = [-0.81, -0.17]
DRS-Memory (MEM)	r(18) = -0.49, p = 0.03 CI = [-0.76, -0.06]	r(18) = -0.63, p = 3x10^-3^ CI = [-0.84–0.26]	r(18) = -0.7, p = 6x10^-4^, CI = [-0.87–0.37]
DRS-Construction (CN)	r(18) = -0.45, p = 0.04 CI = [-0.75, -0.015]	r(18) = -0.61, p = 4x10^-3^ CI = [-0.83, -0.23]	r(18) = -0.61, p = 4x10^-3^, CI = [-0.83,-0.24]
DRS-Conceptualization (CON)	Not significant	r(18) = -0.57, p = 8x10^-3^ CI = [-0.81,-0.18]	r(18) = -0.63, p = 3x10^-3^ CI = [-0.84, -0.26]
DRS-ATT (Attention)	Not significant	Not significant	Not Significant

**Fig 19:**
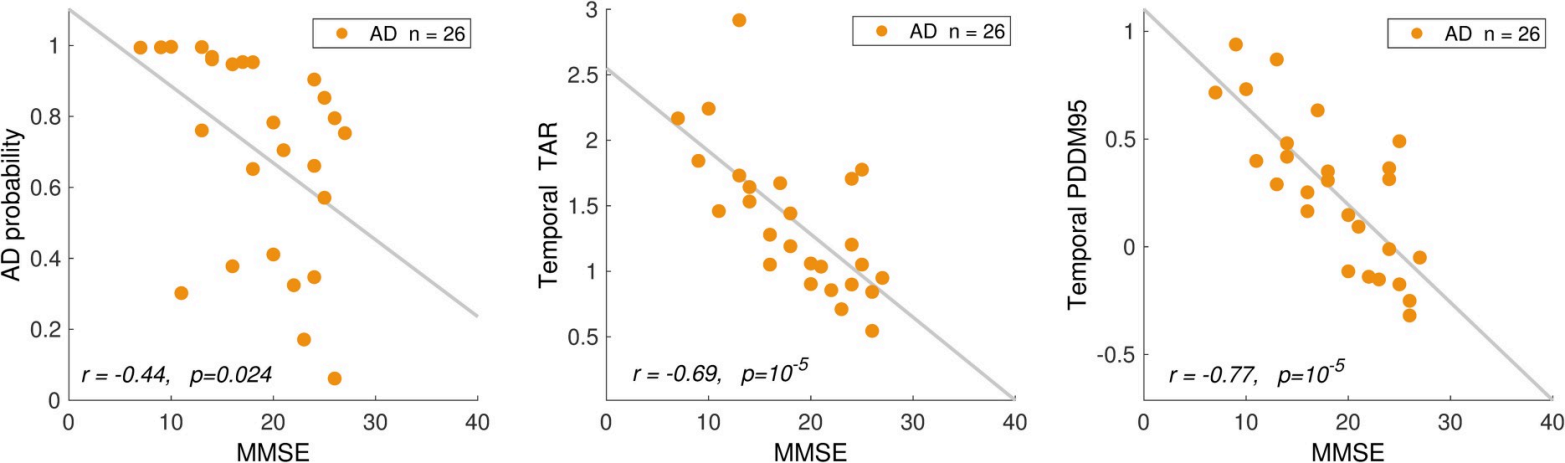
Correlations with clinical scores. Scatterplots showing correlations between MMSE score and (a) probability of AD in AD classifier, (b) TAR at temporal areas, (c) PDDM95 at temporal areas, plotted for all AD participants. PDDM95 has the highest correlation with MMSE.

**Fig 19**A shows scatter plots and Pearson’s correlation coefficients between the EEG measures and MMSE scores for participants in the AD group. Overall probability of AD (computed by the AD classifier) was negatively correlated with MMSE (r(24) = -.44, p = .02). EEG measures of TAR and PDDM averaged at temporal channels were also negatively correlated with MMSE (r(24) = -.69, p = 10^−5^, for TAR) and (r(24) = -.77, p = 10^−5^, for PDDM95). The correlation between PDDM95 and MMSE was highest at channels F7 (r(24) = -.77, p = 3.5x10^-6^)and lowest at channel O2 (r(24) = -.66, p = 2.7x10^-4^). These results are consistent with previous reports [30,36,64,65] reporting the correlation between EEG measures and MMSE scores in AD. However, there was no significant correlation between EEG measures and MMSE scores in the MCI group. The correlations between EEG measures and Dementia Rating Scale (DRS-2) scores are shown in Table 6.

## Discussions and conclusions

This study examined the utility of PSD-based resting state EEG biomarkers to assess neurophysiological changes associated with cognitive decline in Alzheimer’s disease. Specifically, we examined two aspects of these EEG biomarkers, (a) their specificity to pathologic cognitive decline as opposed to normal aging and (b) to propose novel methods for improving the sensitivity of these biomarkers at early stages of the disease observed in individuals with MCI. The results, with respect to the power spectral analyses, agree with prior publications that reported individuals with AD exhibit a progressive slowing of EEG. This slowing is characterized by significant increases in the power of slower frequencies (1–8 Hz), significant decreases in power of faster frequencies e.g. Alpha band (8–13 Hz), and significant slowing of the frequency of the dominant rhythm in the alpha band. Here, we first confirmed these established patterns of EEG in Theta and Alpha bands (and a combined measure defined by the ratio of power at these bands) in our AD group. Second, we studied the MCI group with the hypothesis that they are likely to demonstrate the same patterns with smaller and less significant effects.

Our results showed that while the increased power in low frequencies (Delta and Theta) were indeed observed in the MCI group (albeit much smaller in size), the MCI group did not show a significant change in Alpha power or alpha peak frequency. These results point to a pattern of EEG changes that appears to reflect the progression of the disease that starts with moderate increases in power at the slow frequencies, and only in the later stages of the progression to AD, decreases at the faster frequencies begin to manifest.

In terms of the spatial patterns of EEG changes, the AD group’s differences in both power and coherence (compared to controls) in both Theta and Alpha bands were significant across all areas of the brain. However, for the MCI group, significant differences in Theta band were limited to right temporal, left temporal and left frontal channels with no significant differences in Alpha band. Significant differences in Alpha coherence for the MCI group were detected only at 4 pairs of channels mainly from right temporal to left-temporal and left-frontal areas. In both the MCI and AD groups, the most significant differences (smallest p-values) compared to healthy controls appeared in temporal areas.

The above results suggest that EEG changes in AD may start with localized changes in temporal areas that manifest in the Theta band as increases in both power and coherence, while in the Alpha band only coherence decreases in the same limited areas. These changes may further progress into large and spatially widespread changes in both power and coherence in Theta band (increase) and Alpha band (decrease). This hypothesis is also consistent with the observation that compared to healthy controls, in both the MCI and AD groups, the most significant differences were observed at temporal areas (channels T3,T4,T5,T6). Moreover, the Theta to Alpha ratio had significant correlation with MMSE score of general cognitive abilities that were highest at channel T6. Further research particularly a within-subject analysis in a longitudinal study is needed to confirm this hypothesis that could provide another spatiotemporal characteristic of disease progression using EEG.

Performing the same analyses for 3 separate groups of cognitively normal individuals in different age ranges, confirmed previous reports showing a progressive decrease in slow wave power, specifically in Delta and Theta bands, associated with normal aging [51–53]. Therefore, we concluded that changes in slower frequencies in AD are opposing those of normal aging and hence are specific to pathologic cognitive decline.

### Novel approach to identify subtle EEG abnormalities in MCI

The reported changes in power spectral densities due to AD are well established and significant. In our study, these measures also provided insights into possible spatiotemporal distribution patterns of disease progression. However, these changes are shown to be difficult to detect in early stages of AD or in individuals with MCI. The EEG abnormalities might be subtle and limited both in terms of spatial distribution across the scalp and temporal distribution across recording time. We demonstrated this by the observation that EEG changes in the MCI group were smaller and localized, compared to the AD group. Therefore, we introduced a novel method based on the distance between distribution of EEG power (across time) at the Theta and Alpha bands as opposed to the average of the Theta to Alpha ratio across time. By preserving the PSD data at the epoch level, this measure remained sensitive to changes in EEG that may not be consistent across the recording session but rather may happen at isolated time points. Our proposed measure using this approach did not outperform the standard Theta to Alpha ratio (TAR) when comparing the AD group to age matched controls. However, for the MCI group it resulted in significant differences that were more widespread across the scalp, with larger effect size and smaller p-value.

### Limitations of this study

This study has few limitations as follows. (a) AD and MCI participants in this study were selected based on clinical neuropsychological diagnosis, without confirmation by neuropathological diagnostic biomarkers. These diagnostic methods are still the most common standards of clinical practice with variations in criteria at different clinical sites. This may result in heterogenous study groups with varying degrees of disease progression and underlying pathology. This is particularly the case for the MCI group, as they may or may not progress into AD based on their underlying pathology. (b) While we demonstrated the specificity of the proposed EEG biomarkers to pathologic cognitive decline compared to normal aging, the results do not provide support for the specificity of these markers in AD compared to other neurodegenerative disease. For example, AD, Parkinson’s disease dementia (PDD) and Lewi Body dementia (LBD) may have EEG biomarkers in common. (c) While this study demonstrated the association between EEG biomarkers and disease progression, the mechanistic relation between the basic elements in the triad of protein pathologies, synaptic losses/dysfunctions and EEG abnormalities were outside of the scope of this work. (d) This study was focused on group analysis rather than assessment of disease progression at an individual level. However, for clinical translation of these methods at an individual level, a high level of sensitivity and specificity is needed for MCI. In this study, it was not possible to assess these methods at an individual level due to heterogenous nature of the MCI group. However, the high correlation between MMSE score and EEG measures in the AD group supports their utility even at an individual level in later stages of AD. Future work to better assess the performance of these methods at an individual level may include: using more homogenous study groups in terms of disease progression, including clinicians’ expert judgment in artifact rejection and data selection before automated quantitative analysis, and incorporating a more comprehensive approach that includes both EEG and non-EEG clinical. In applications such assessment of treatment effect in clinical trials for drug discovery, group analysis might be enough to demonstrate the treatment effect.

#### The rationale for use of EEG in clinical research and clinical trials

There are multiple approaches toward disease modifying therapeutic intervention in AD clinical trials. Many drug development efforts target amyloidosis and tauopathy [3] focusing on protein pathologies. Therefore, primary outcome measures may focus on CSF-based pathological biomarkers along with neuropsychological scores to assess the changes in syndromes.

However there is an alternative/complementary treatment strategy that focuses on synaptic dysfunction as the biological mechanism that links protein pathologies to disease symptoms [66–70]. EEG-based biomarkers are more directly correlated with such neural dysfunctions and might be a better candidate for assessing disease progression. Furthermore, there have been evidences from animal models in mice, suggesting that neural abnormalities (increased neural synchrony) may occur even before prominent amyloid plaque deposition [71,72].

Converging lines of evidence [11,26] support the utility of EEG as an early biomarker of disease progression in AD. EEG biomarkers for AD can be categorized as “topographical biomarkers” suitable for disease progression while “pathophysiological biomarkers” are used for diagnosis [10]. Topographical biomarkers such as EEG and FDG-PET are more directly associated with neural dysfunctions and can identify regional distribution of AD pathology.

EEG is non-invasive, inexpensive, and relatively simple to implement for large scale clinical research. The rationale for use of EEG in clinical trials is also strengthened by the finding that PSDs in EEG have proven remarkably stable within individuals [73–77] particularly during mental tasks [78]. Therefore, in clinical trials and studies with a within-subject design, EEG biomarkers such as the ones discussed in this manuscript could be used to detect and monitor neurophysiological changes that may be associated with treatment or intervention.

## Supporting information

S1 Fig Topographical maps of the Pearson’s correlation coefficient between EEG measures PDDM95 (left) and TAR (right) at each channel versus cognitive score MMSE (top) and DRS-MEM bottom. (TIF)

S2 Fig Topographical maps of the Alpha peak frequency (in Hz) in AD and HC3 groups. (TIF)

S1 Table Global average PSD measures for all frequency bands in all participant groups. (DOCX)

S1 File (PDF)
