# Supplementary figures and images for: Resting state EEG biomarkers of cognitive decline associated with Alzheimer’s disease and mild cognitive impairment
Source: PLoS One. 2021 Feb 5;16(2):e0244180. doi: 10.1371/journal.pone.0244180 (PMC7864432; doi:10.1371/journal.pone.0244180)

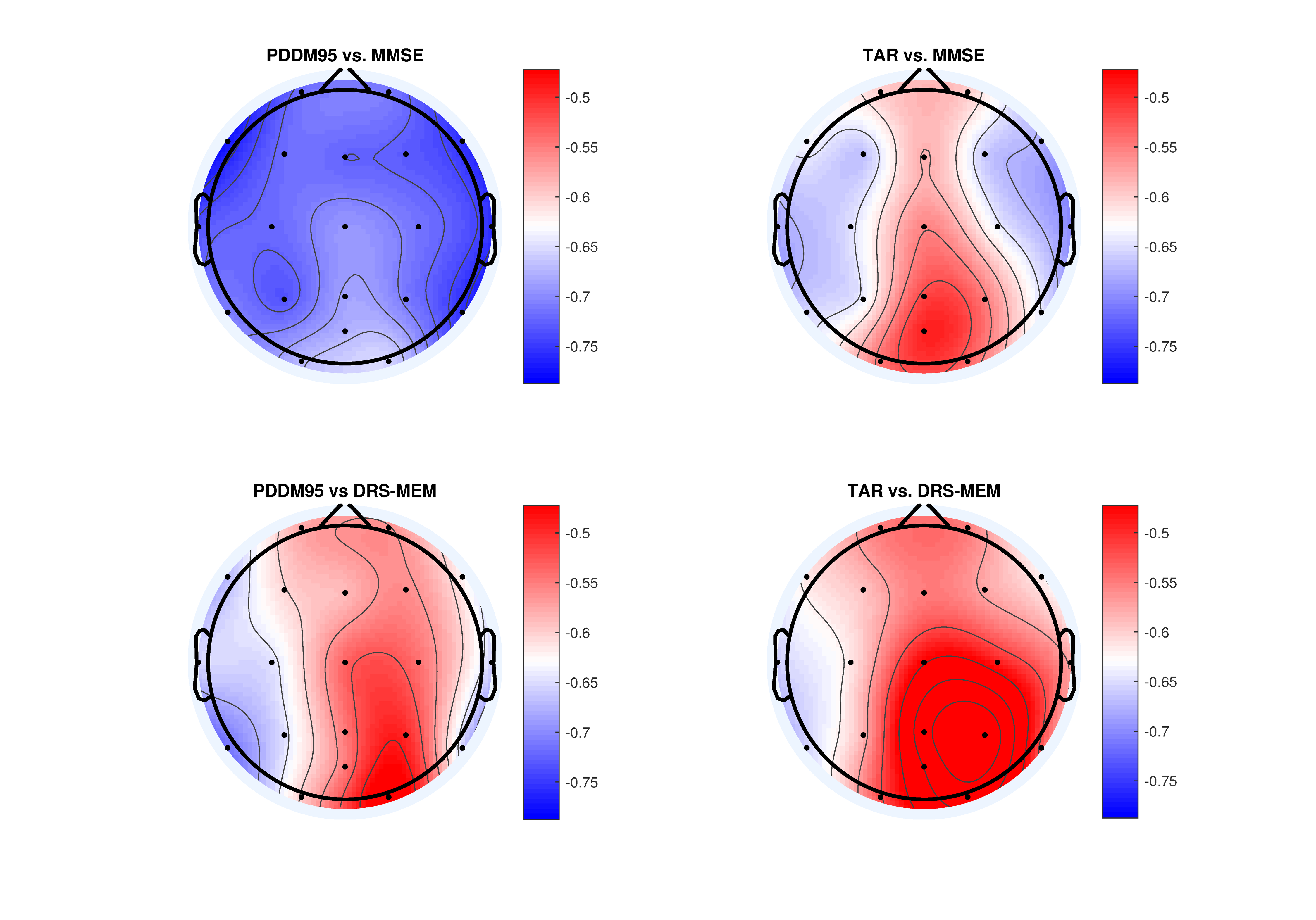

Supplement: S1 Fig — (TIF) [file pone.0244180.s001.tif]

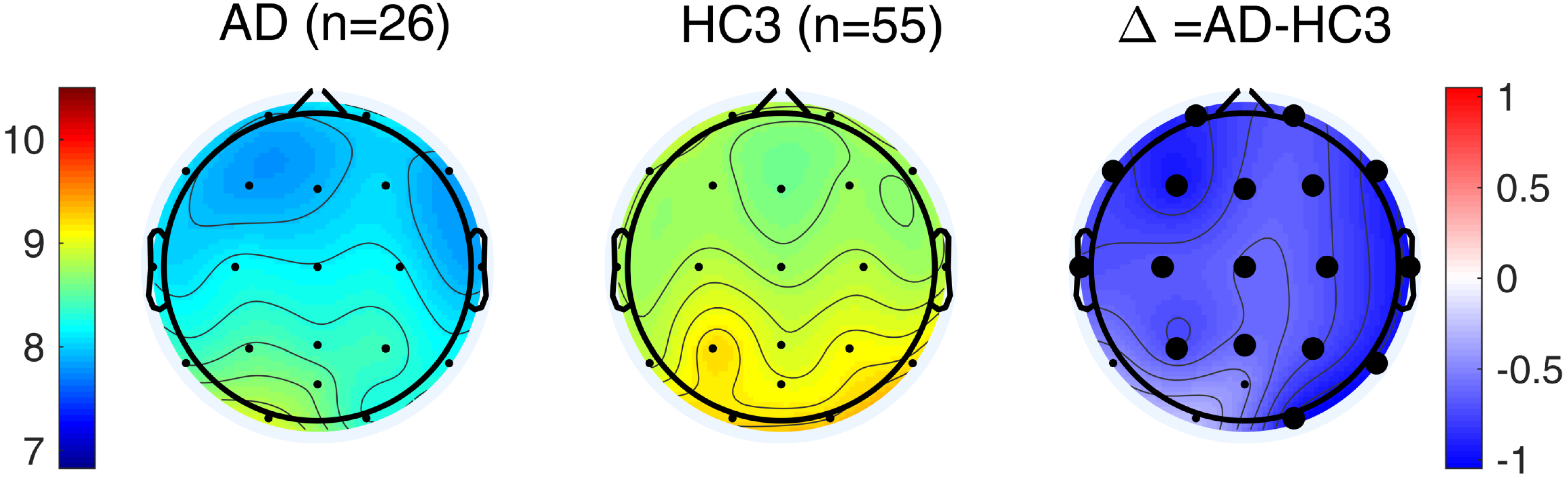

Supplement: S2 Fig — (TIF) [file pone.0244180.s002.tif]
