# Supplementary material for: Resting state EEG biomarkers of cognitive decline associated with Alzheimer’s disease and mild cognitive impairment
Source: PLoS One. 2021 Feb 5;16(2):e0244180. doi: 10.1371/journal.pone.0244180 (PMC7864432; doi:10.1371/journal.pone.0244180)
Supplement: S1 Table — (DOCX) [file pone.0244180.s003.docx]

**S1 Table. Global average PSD measures for all frequency bands in all participant groups.**

| **EEG measure (mean ± SEM)** | **HC1** | **HC2** | **HC3** | **MCI** | **AD** |
| --- | --- | --- | --- | --- | --- |
| Overall-Delta_1_3 | 2.56±0.021 | 2.47±0.027 | 2.39±0.031 | 2.48±0.043 | 2.63±0.082 |
| Overall-ThetaSlow_3_5 | 2.38±0.023 | 2.27±0.031 | 2.16±0.035 | 2.25±0.043 | 2.39±0.08 |
| Overall-ThetaFast_5_7 | 2.32±0.028 | 2.22±0.043 | 2.18±0.047 | 2.3±0.056 | 2.35±0.08 |
| Overall-ThetaTotal_3_7 | 2.37±0.025 | 2.28±0.037 | 2.21±0.041 | 2.32±0.049 | 2.4±0.079 |
| Overall-AlphaSlow_8_10 | 2.58±0.044 | 2.51±0.058 | 2.58±0.061 | 2.62±0.066 | 2.38±0.084 |
| Overall-AlphaFast_10_13 | 2.44±0.038 | 2.31±0.049 | 2.3±0.049 | 2.29±0.049 | 2.1±0.07 |
| Overall-AlphaTotal_8_13 | 2.5±0.039 | 2.41±0.051 | 2.45±0.054 | 2.48±0.057 | 2.25±0.076 |
| Overall-Beta_13_30 | 1.65±0.02 | 1.72±0.033 | 1.82±0.035 | 1.76±0.035 | 1.76±0.061 |
| Overall-Gamma_25_40 | 1.2±0.022 | 1.29±0.036 | 1.43±0.044 | 1.39±0.046 | 1.54±0.076 |
| Overall-BetaSlow_13_20 | 1.8±0.022 | 1.86±0.035 | 1.95±0.035 | 1.89±0.036 | 1.83±0.058 |
| Overall-BetaFast_21_30 | 1.44±0.019 | 1.51±0.033 | 1.63±0.038 | 1.58±0.039 | 1.66±0.068 |
| Overall-Delta_1_3_Rel | 0.08±0.003 | 0.07±0.003 | 0.06±0.003 | 0.07±0.004 | 0.09±0.008 |
| Overall-ThetaSlow_3_5_Rel | 0.05±0.002 | 0.05±0.002 | 0.04±0.002 | 0.04±0.002 | 0.05±0.005 |
| Overall-ThetaFast_5_7_Rel | 0.04±0.001 | 0.04±0.002 | 0.04±0.002 | 0.04±0.003 | 0.05±0.005 |
| Overall-ThetaTotal_3_7_Rel | 0.05±0.001 | 0.04±0.002 | 0.04±0.002 | 0.04±0.002 | 0.05±0.004 |
| Overall-AlphaSlow_8_10_Rel | 0.09±0.004 | 0.08±0.005 | 0.09±0.005 | 0.09±0.006 | 0.05±0.006 |
| Overall-AlphaFast_10_13_Rel | 0.06±0.002 | 0.05±0.003 | 0.05±0.002 | 0.04±0.002 | 0.03±0.003 |
| Overall-AlphaTotal_8_13_Rel | 0.07±0.002 | 0.06±0.003 | 0.06±0.003 | 0.06±0.003 | 0.04±0.004 |
| Overall-Beta_13_30_Rel | 0.01±0 | 0.01±0.001 | 0.01±0.001 | 0.01±0.001 | 0.01±0.001 |
| Overall-Gamma_25_40_Rel | 0±0 | 0.01±0.001 | 0.01±0.001 | 0.01±0.001 | 0.01±0.001 |
| Overall-BetaSlow_13_20_Rel | 0.01±0 | 0.02±0.001 | 0.02±0.001 | 0.02±0.001 | 0.01±0.001 |
| Overall-BetaFast_21_30_Rel | 0.01±0 | 0.01±0.001 | 0.01±0.001 | 0.01±0.001 | 0.01±0.001 |
| Overall-TBR | 2.48±0.064 | 2.1±0.091 | 1.74±0.083 | 2.07±0.099 | 2.46±0.252 |
| Overall-sTBR | 2.51±0.07 | 2.1±0.093 | 1.65±0.075 | 1.95±0.088 | 2.49±0.276 |
| Overall-sTfBR | 3.25±0.105 | 2.76±0.14 | 2.12±0.111 | 2.49±0.13 | 2.88±0.33 |
| Overall-TAR | 1.01±0.038 | 0.97±0.044 | 0.83±0.035 | 0.92±0.045 | 1.34±0.116 |
| Overall-TGR | 4.32±0.143 | 3.65±0.206 | 2.9±0.174 | 3.34±0.197 | 3.39±0.391 |
| Overall-sTGR | 4.33±0.148 | 3.6±0.194 | 2.74±0.158 | 3.13±0.175 | 3.42±0.413 |
| Overall-AlphaPeak Frq. (Hz) | 9.82±0.099 | 9.59±0.133 | 9.04±0.107 | 8.79±0.141 | 8.33±0.319 |
| Overall-AlphaPeakPower | 2.76±0.046 | 2.65±0.06 | 2.67±0.065 | 2.72±0.069 | 2.61±0.089 |

* mean values smaller than 0.01 and standard error of the means smaller than 0.001 have been rounded to zero.
TBR: Theta to Beta ratio, sTBR: SlowTheta to Beta ratio, sTfBR: slow Theta to fastBeta ratio, TAR: Theta to Alpha ratio, TGR: Theta to Gamma ratio, sTGR: slowTheta to Gamma ratio.
