# Supplementary material for: Resting state EEG biomarkers of cognitive decline associated with Alzheimer’s disease and mild cognitive impairment
Source: PLoS One. 2021 Feb 5;16(2):e0244180. doi: 10.1371/journal.pone.0244180 (PMC7864432; doi:10.1371/journal.pone.0244180)
Supplement: S1 File — (PDF) [file pone.0244180.s004.pdf]

EEG analytics: benefits and challenges of data driven EEG biomarkers for neurodegenerative diseases

Amir H. Meghdadi, Marija Stevanović Karić, Chris Berka

Abstract— **Automated quantitative analysis of EEG data is ubiquitous in clinical neurophysiology research. However, these methods have not been fully adopted in medical practice, specifically to inform clinical diagnosis of neurological disorders. In this paper, some of the benefits and challenges of using these techniques as biomarkers for disease indication and progression are discussed. Examples of baseline EEG (resting state) data acquired with the same system and software from patients diagnosed with four types of neurodegenerative diseases are presented and compared with healthy controls.**

Overall power spectral density analyses showed clear significant differences in EEG for patient sub-types including enhanced theta power in dementia patients (Alzheimer's and Lewy-body dementia), enhanced beta power in Parkinson's patients (with or without dementia) and reduced Alpha power in Alzheimer's dementia.

To assess the discriminating power of resting state EEG spectral measures particularly for differential diagnosis at an individual level, a binary classifier was designed to classify EEG data across conditions after feature dimensionality reduction (PCA). The results were evaluated using the area under classifier's ROC curve (AUC). On average, data acquired during eyes-closed resting state resulted in better classification than eyes-open. The highest classification performance (against healthy control) was obtained for Alzheimer's dementia (AD) and Lewy-body dementia (LBD) with AUC=0.80 in both cases. The least accurate classification results were obtained for Mild Cognitive Impairments (MCI) group. The challenges of using resting state EEG for characterization of MCI is discussed and other examples of EEG biomarkers for MCI based on event-related-potentials in a cognitive test of visual memory are presented.

An argument is made for adequate sensitivity/specificity of the current state-of-the-art in data-driven EEG analytics as secondary end-points in clinical trials. However, routine clinical diagnostic at an individual level may require a paradigm shift incorporating techniques of precision medicine and big data analytics. Multi-modal public datasets (including EEG and other imaging/clinical data) and adopting standards/protocols for sharing anonymized data between researchers could likely facilitate development of more reliable biomarkers in clinical practice.

I. INTRODUCTION

Electroencephalography (EEG) is a routine practice in clinical evaluation of epilepsy and several other clinical indications [1]. However, despite the mounting evidence for EEG signatures of neurodegenerative diseases such as Alzheimer's, [2]–[7], EEG is not part of the standard practice of clinical diagnosis in these indications. Traditional "reading" of EEG requires a board-certified expert to visually examine the record and to make an assessment in the context of patient's individual clinical presentations [1]. However EEG signatures of neurodegenerative diseases that can be detected by visual inspection (such as diffuse slowing [8] in advanced AD), may not be visible in prodromal and early stages of disease [9], [10].

In contrast, data-driven and computer assisted analysis of EEG using data analytics, statistics and machine learning may be able to detect and quantify meaningful information that is not apparent in visual inspection. Unlike manual EEG reading, automated data analysis at its basic level relies on statistical properties of the data.

The use of quantitative methods in EEG analytics was extensively assessed in a 1997 report published by American Academy of Neurology [11]. In that report these techniques were identified to be potentially useful in some applications while investigational in other domains. For example, using frequency analysis for dementia was considered possibly useful and classified as "*positive recommendation*" if used in expert hands. However, in the past 20 years there has been considerable progress in automated EEG analytics [12] [13] especially in neurodegenerative diseases ([6], [14]). Moreover, it is believed that less-expensive and less-invasive biomarkers for diseases such as Alzheimer's are needed [15] in order to use biomarkers beyond research and in clinical practice.

The main contribution of this paper is to provide a preliminary demonstration of the discriminating power of resting-state EEG biomarkers in differential diagnosis of neurodegenerative diseases and discuss the challenges associated with using these methods in clinical practice. Results from four different patient populations as well as a healthy control group collected using the same data acquisition system, software, protocols and analysis pipeline will be presented. Limitations and challenges of these techniques will be discussed. The rest of this paper is as follows. In section II, the utility and reliability of these methods will be discussed. Section III lists the common acquisition protocols. Section IV shows example of EEG biomarkers in selected patient populations and the results of a linear classifier on resting state baseline EEG is shown. The limitations of the current methods especially for MCI group will be discussed and the results of event-related-potential analysis for a cognitive memory task will be presented. Section V concludes the paper discussing how further improvement of specificity and sensitivity in data-driven EEG biomarkers may require big data analytics in large and multi modal data sets to build complete models that include both EEG and other relevant clinical data.

*A.M. M.S.K. and C.B. are with Advanced Brain Monitoring, CA, 92008 USA (phone: 760-720-0099; e-mail: amir@b-alert.com).

II. UTILITY, RELIABILITY AND STANDARDIZATION

EEG as a biomarker that is based on direct measure of cortical neural activity has many benefits including non-invasive, low-cost and portable equipment. However, there are challenges in (a) acquiring reliable metrics, and (b) developing a standard scale for EEG metrics that can be used as a normative baseline regardless of the equipment and analysis techniques. Raw recorded data often contain noise and are contaminated with artifacts such as those generated by eye blink, muscle movements or environmental noise. Movement artifacts in particular can make it difficult to acquire high quality signals in young children, babies and certain patient populations with severe physical or emotional syndromes. Artifact decontamination normally requires manual inspection and expert's judgments on excluding parts of the signal or its components (in the case of independent component analysis [16]). This will reduce reproducibility and inter-rater reliability. However, automated artifact rejection and decontamination methods have been proposed to automatically detect and remove artifacts based on analytical methods (e.g. [17]) or component classification trained by labels obtained through crowdsourcing [18]. Some EEG protocols require simultaneous EEG and cognitive tasks to elicit event related potentials (ERPs). EEG recording concurrent with cognitive tasks can be impeded by severe cognitive decline, where the subject is either unable to understand and perform the tasks or unable to continue due to frustration. Examples of such cases will be given in the following sections. In order for EEG biomarkers to be applied as standard practice in the clinic, EEG measures need to be normalized and standardized to be useful as metrics of disease indication and progression and translatable across clinicians and researchers. However, these quantified metrics are usually highly dependent on the analysis parameters and hardware and software specifications making it difficult to build independent normative databases.

III. ACQUISITION PROTOCOLS AND ANALYSIS METHODS

EEG recording can be classified as one of the following two types (a) baseline resting state EEG with eyes-open or eyes-closed or (b) EEG recording concurrent with some sensory stimulation or provocation (e.g. photic stimulation or hyperventilation), steady state evoked potentials (SSEP) or event related potentials (ERPs) during cognitive tasks.

A. EEG during resting state

Recording baseline EEG activity during resting state with eyes-open or eyes-closed is the simplest form of EEG acquisition. Analysis methods may include power spectral density analysis, functional connectivity analysis and synchrony measures (e.g. spectral coherence, Granger causality, phase synchrony indices, etc. [19]–[21]). Source localization by solving the inverse-problem or independent component analysis and dipole fitting are also common but generally require large number of channels.

B. Event-related potential analysis during cognitive tasks

Analysis of evoked EEG patterns in response to stimuli requires more complicated testing protocols with stimulus presentation software synchronous with EEG recording and relies on repeated stimulus presentation and averaging EEG signal during trials of the same type.

IV. EEG BIOMARKERS FOR NEURODEGENERATIVE DISEASES

In this section the discriminatory power of resting state PSD measures as EEG biomarkers for four different types of neurodegenerative diseases is evaluated.

A. Patients demographic

Under IRB approval, a total of 124 healthy controls and 89 patients with different types of neurodegenerative diseases including Mild Cognitive Impairment (MCI), Alzheimer's disease (AD), Parkinson's disease without dementia (PD), Lewy body dementia (LBD, including Parkinson's disease dementia and dementia with Lewy bodies) were selected for this study (Table I).

TABLE I. PATIENT'S DEMOGRAPHIC

Code	Condition	N	Age (mean)
HC	Healthy controls	124	40-84 (57.4)
MCI	Mild cognitive impairment	36	53-88 (70.6)
AD	Alzheimer's disease	22	58-90 (72.2)
PD	Parkinson's disease	22	52-76 (62.8)
LBD	Lewy body dementia*	9	66-78 (72.4)

* Constitutes either Dementia with Lewy bodies type (DLB) or Parkinson's disease Dementia (PDD)

B. Equipment and Data Collection

This data set was collected across various studies all recorded using the Stat X24 wireless EEG system (Advanced Brain Monitoring, Carlsbad, CA, USA). Stat X24 is an FDA-cleared battery-powered, lightweight, wireless EEG system that acquires 20 channels of EEG according to 10-20 standards and referenced to linked mastoids. It uses passive Ag/AgCl electrodes with flexible, flat cables printed on PET (Polyester) strips. The sampling rate is 256 Hz and the low and high cut off frequencies of the amplifier is 0.1 Hz and 100 Hz, respectively. Data are amplified and digitized (using a 16-bit A/D) in the wearable unit on the headset and then transmitted wirelessly via Bluetooth to a host computer.

C. Methods: EEG artifact decontamination and classification based on power spectral density

EEG data were recorded during both 5-min eyes-open and 5-min eyes-closed sessions in resting state. Data was bandpass filtered (1-40Hz). Independent component analysis (ICA) was performed using EEGLAB software [16]. Artifact decontamination was performed using ICLabel toolbox to reject components classified as having sources other than brain (e.g. eye blinks, EMG, etc.). ICLabel uses a classifier that is pre-trained by thousands of labelled components obtained through crowdsourcing [18]. Power spectral densities were computed using Fast Fourier Transform with Kaiser window on 1 second long windows with 50% overlap. The total power in each frequency bin 1 to 49 Hz and each frequency bandwidth (Delta, Theta, Alpha, Beta and Gamma) at each one of the 20 channels were computed for each epoch and were averaged across all the epochs during each session resulting in 2140 features that are highly correlated. Dimensionality reduction was performed using principal component analysis (PCA) through singular value decomposition. The first k components accounting for more than 98% of the total variance were selected for downstream analyses with the remaining components rejected.

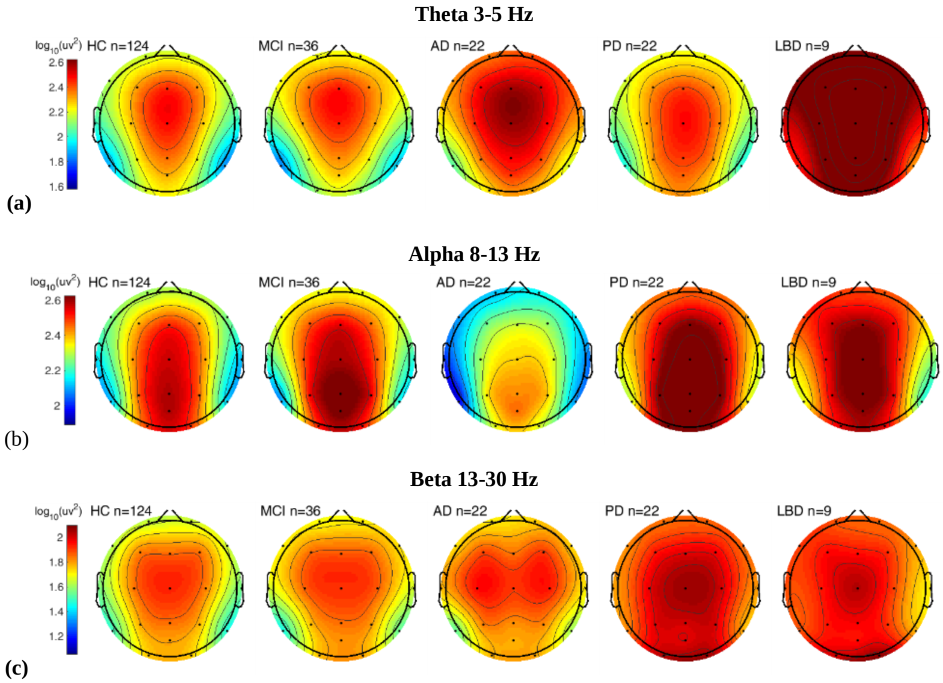

Figure 1. Topographical maps of group average (not age matched) EEG power during resting state eyes closed at 3 frequency bands (a) Theta (3-5 Hz), (b) Alpha (8-13 Hz) and (c) Beta (13-30 Hz) in each patient population. Some of the significant findings are reduced Alpha power in AD patients, enhanced theta power in AD, LBD (PDD and DLB) and enhanced beta power in PD and PDD.

A total of 10 linear classifiers were trained and tested for each pair of groups (patients or healthy controls). For each classifier the subjects were age matched to include patients in the same age range. Each classifier was trained using a Linear Discriminant Analysis (LDA) providing a linear combination of features (principal components) that best separate the two classes (according to Fischer criterion for separability). The area under the curve (AUC) for the receiver operating characteristic (ROC) curve was computed as a measure of classifier performance. The classifiers were subsequently cross validated by leave-one-subject-out (LOSO) cross validation method and the AUC was reported for both auto-validation (tested on training set) and cross validation method to indicate the overfitting for each classifier. Due to significant imbalance in the number of patients in each group (class sizes), a bootstrapping technique was used as follows. For each classification, *n* subjects from the largest class were randomly selected where *n* is the number of subjects in the smaller class and a classifier was trained and tested on the two equal size classes. The process was repeated 100 times and the classification results were averaged across the 100 iterations.

D. Results: Resting state PSD EEG biomarkers

Comparison of the group averages for patients and healthy controls showed a significant increase in theta and decrease in alpha power in AD group [7], a significant increase in beta power in Parkinson's group, and a significant increase in Theta power for patients with LBD (either Dementia with Lewy Bodies or Parkinson's disease dementia). These findings were consistent with previous published reports [6], [22]. Other significant changes that are recently reported in neurodegenerative disease include a strong and significant reduction in Alpha power for patients with Huntington's disease [23].

Figure 1 shows average topographical maps for three frequency bands (Theta, Alpha and Beta) plotted for each patient population during eyes-closed session. Although there are significant differences between group averages, these measures may not have acceptable sensitivity and specificity for clinical diagnosis at individual patient level. Therefore, we designed binary classifiers to evaluate the discriminatory power of these biomarkers in differential diagnosis of neurodegenerative diseases. Absolute and relative spectral powers at each 1-Hz binned frequency range and standard bandwidths at each channel were used to train a classifier according to the method in section C. Table II shows the classification results for each pair of neurodegenerative groups for eyes-closed and eyes-open, respectively. The AUC of the classifiers after cross validation is shown in bold. On average the eyes-closed data resulted in better classification performance. These results for AD, LBD, and PD groups are promising and comparable with previously published reports in the literature [24]–[28]. Full description of the classification results including sensitivity and specificity for each indication versus healthy controls are listed in Table IV. The ROC curves (after bootstrapping) for all classifiers are shown in Figure 2. MCI cannot be reliably classified against healthy controls after cross-validation. This classifier has the lowest AUC when tested on the training data (auto-validation; AUC= 0.89; sensitivity=79.67; specificity=84.36; ppv=83.70; npv=80.72) and a cross validation AUC that drops to 0.61 (sensitivity=59.60; specificity=60.15; ppv=62.14; npv=59.24). However, this is to be expected mainly because of heterogeneity of the MCI cohort with respect to both the cognitive impairments and

TABLE II EYES-CLOSED (LEFT) AND EYES-OPEN (RIGHT) RESTING STATE CLASSIFIERS. AUC FOR CROSS VALIDATION IS SHOWN IN BOLD AND AUC FOR AUTOVALIDATION IS SHOWN IN PARENTHESES.

Eyes-Closed

	HC	MCI	AD	PD	LBD
HC		**0.61** (0.89)	**0.80** (0.98)	**0.60** (0.94)	**0.80** (1.00)
MCI	**0.61** (0.89)		**0.59** (0.90)	**0.64** (0.93)	**0.75** (0.98)
AD	**0.80** (0.98)	**0.59** (0.90)		**0.78** (0.99)	**0.77** (0.99)
PD	**0.60** (0.94)	**0.64** (0.93)	**0.78** (0.99)		**0.63** (0.96)
LBD	**0.80** (1.00)	**0.75** (0.98)	**0.77** (0.99)	**0.63** (0.96)	

Eyes-Open

	HC	MCI	AD	PD	LBD
HC		**0.52** (0.85)	**0.73** (0.98)	**0.69** (0.98)	**0.69** (1.00)
MCI	**0.52** (0.85)		**0.48** (0.89)	**0.76** (0.98)	**0.65** (0.98)
AD	**0.73** (0.98)	**0.48** (0.89)		**0.76** (0.97)	**0.63** (0.99)
PD	**0.69** (0.98)	**0.76** (0.98)	**0.76** (0.97)		**0.64** (0.97)
LBD	**0.69** (1.00)	**0.65** (0.98)	**0.63** (0.99)	**0.64** (0.97)	

pathological changes (e.g. longitudinal studies show that MCI patients may or may not eventually convert to AD or to other dementias).

TABLE IV SENSITIVITY AND SPECIFICITY OF CLASSIFIERS FOR EACH GROUP VS. HEALTHY CONTROL USING EYES-CLOSED DATA

	MCI	AD	PD	LBD
Sensitivity	59.60	74.41	57.89	77.31
Specificity	60.15	76.27	57.94	79.09
PPV*	62.14	77.77	58.39	80.44
NPV**	59.24	73.24	58.04	76.62

* Positive predictive value, ** Negative predictive value

These results are also consistent with previous studies classifying MCI and AD patients using structural MRI to classify these groups based on structural alternations associated with AD and prodromal AD. In [29], measures derived from inter-regional covariation of cortical thickness in structural-MRI images, showed a similar trend with challenging classification for healthy versus MCI (including those with and without conversion to AD).

E. *Methods: image recognition memory test*

An ERP task designed to evaluate image recognition memory was included in the EEG protocol for patients in MCI, AD, LBD and a subset of HC groups. The task involves presenting participants with a series of 20 images (called Target stimuli) to memorize in a training phase (each presented twice in random order on the screen for 1.25 seconds with a 3 seconds inter-stimulus-interval). In the testing phase, a total of 100 images (a collection of the 20 Target images randomly interspersed with 80 new NonTarget images) are presented on the screen each for 1.5 seconds with a 3 second inter-stimulus interval. The participants were instructed to respond with Yes or No, using the keyboard to indicate if the image is Target or NonTarget. EEG signals were recorded simultaneously synchronized with stimulus presentation using External Synching Unit (ESU) hardware included in ABM's X24 EEG acquisition system. Raw signals were filtered between 0.1 and 40 Hz. For each event type, EEG data were epoched from 1 second before until 2 seconds after the stimulus onset. The baseline was adjusted using data from 100ms before the stimulus onset. Trials were rejected if the absolute value of EEG amplitude in any channel during a window of -50 ms to +750 ms (compared to the stimulus onset) was larger than a threshold level of 100 microvolts. Artifacts were removed using the Independent component analysis (ICA) approach described in section C above. Moreover, epochs with abnormal spectra (with spectrum 35dB higher or lower than the baseline in the

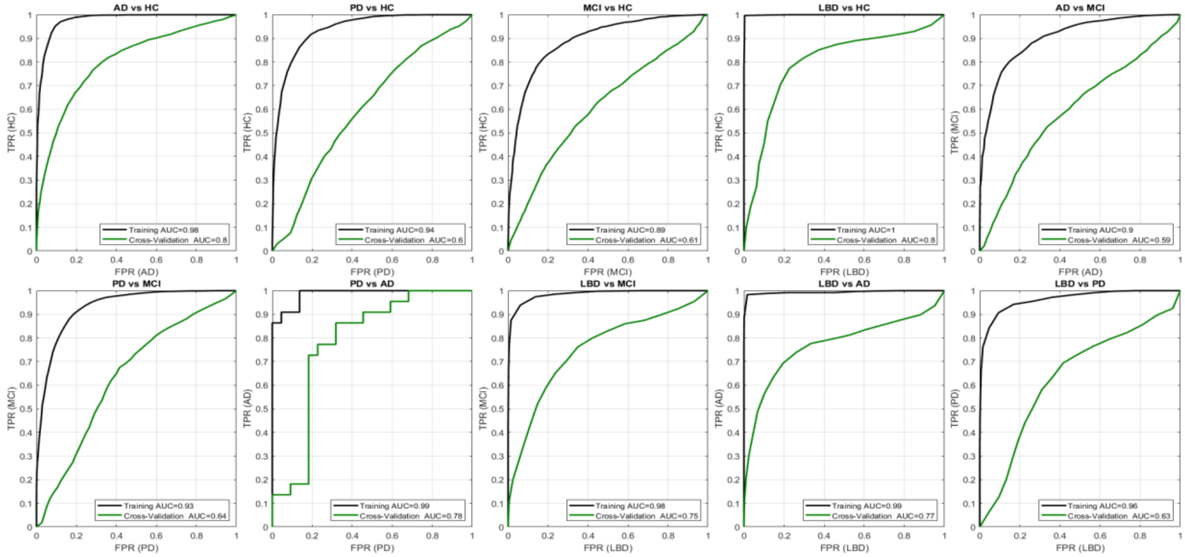

Figure 2. ROC curves for all the classifiers, plotted for both auto-validation (in black) and cross-validated and bootstrapped (in green)

frequency range of 20-30 Hz) were also excluded using EEGLAB. ERPs for each subject were averaged across included trials (minimum 10 trials of the same type (Target or NonTarget) for each participant.

F. Results: image recognition memory test

Although the image recognition task was preceded with a set of instructions and included a practice session, most patients in the late stages of AD and LBD were not able to perform or complete these tasks due to severe cognitive decline or frustration and inability to understand the instructions. Further analysis of the available data indicated that most patients with Mini Mental State Exam (MMSE) scores below 20 were unable to complete the tests. However most patients in MCI group successfully completed the memory task. The grand average ERP results (plotted in Figure 3 for channel Cz as an example) showed significant delay (~40 ms) in early components of the waveforms in MCI. A binary classifier was designed using the same approach described in section C with input variables consists of ERP measurements as follows. Late positive potential (LPP) measured by averaging the amplitude of ERP within a window of [400-800] ms post stimulus. Maximum amplitude (peak) within a window [150-250] ms post-stimulus, latency of the above peak and the latency of the minimum amplitude within [250-350] ms post-stimulus measured for each of the 20 channels, resulting in 80 features in total. The resulting classifier has an AUC=0.71 (sensitivity=68.52; specificity=67.35; ppv=68.02; npv=68.14).

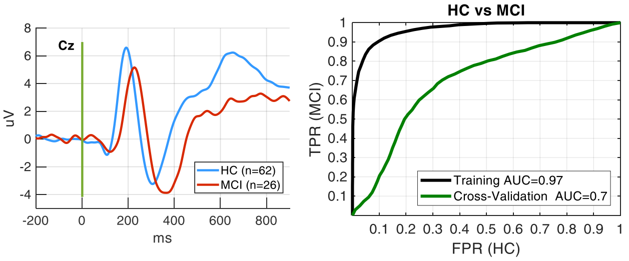

Figure 3 Grand average ERP waveform (Left) for MCI patients shows a significant delay in early components and reduced amplitude of the late component compared to healthy controls. The ROC curve of the classifier shows improved performance compared to resting state data

G. Discussion

The effect of neurodegeneration on EEG measures is demonstrated in numerous studies including the results presented in the current paper. In many cases, the association between EEG abnormalities and neuropathology of the disease is also reported; for example in Alzheimer's [22] or Parkinson's [30]. In the previous sections the discriminating power of basic resting state spectral measures were evaluated across a range of neurodegenerative disease. While improving these results is possible through more advanced classifier design and larger data sets, the present work aimed at exploring the reliability of these biomarkers via using basic spectral measures with a linear classifier to identify a) which frequency bands have more discriminating power and b) which differential diagnoses are more challenging. For example, the preliminary results show that theta band measures are more affected (enhanced) in patient with dementia (*i.e.* AD and LBD) whereas beta band measures are enhanced in patients with Parkinsonism (PD and LBD). The difference between auto-validation and cross validation of the classifiers shows the degree of overfitting which was very high for MCI patients. It is worth noting that while other classifiers had near 100% performance on training data (auto-validation; to be expected) MCI vs HC, MCI vs AD and MCI vs PD classifiers all had lower auto-validation performance that also dropped considerably after cross-validation and bootstrapping. These results might indicate the inherent heterogeneity of MCI population as a group which is not suitable for a binary classification. For example MCI can include a wide range of patients, those with and/or without AD pathology [31]. A longitudinal study is needed to identify the subset of MCI patients that will progress into AD. Using data from event related potentials (ERP) during a cognitive test of memory, MCI group showed significant delay in ERP response and its classification was slightly improved. However, these tests appear to be more sensitive to parameters of the protocol and the patient population which makes it more difficult to be established as routine clinical practice. For example, a similar previous ERP study using the same protocol with different inter-stimulus-interval [4] showed only reduced amplitude and no delay in ERP components in MCI.

V. CONCLUSIONS

EEG biomarkers have been successfully used in both research and clinical trials relying on the presence of statistically meaningful differences and effects between experimental conditions or patient populations. The fundamental challenge in clinical use of these biomarkers is hypothesized here to be related to oversimplification in definition of these indications (*i.e.* binary classification of an individual into two heterogeneous groups of patients and healthy controls). An evidence supporting this hypothesis is the high discriminating power of these biomarkers in indications such as Huntington's disease where the diagnosis can be easily confirmed with DNA determination as opposed to diagnosis of MCI or AD. For example, in [32], the authors were able to train a classifier with a high performance (AUC = 0.9) to classify HD patients vs healthy controls. In the case of Alzheimer's disease and MCI, while clinical diagnostic guidelines exist, proper diagnosis criteria is still being discussed in the research community and has been shifted towards a neuropathological as opposed to a syndromal definition [15], [33]. For example, the patients in the present paper were diagnosed based on clinical symptoms and may not be a homogenous group especially in MCI. These limitations become more significant in the absence of a sensitive ground truth for disease progression that is based on both cognitive decline (symptoms) and pathological changes (CSF/imaging biomarkers). For example while MMSE measures of cognitive decline were reported to be correlated with EEG biomarkers in AD [7], no correlation was observed in MCI patients. Further improvement in these EEG biomarkers could benefit from exploratory multimodal big data analytics that include all aspects of disease progression (symptomal and pathological) and individual patient information such as medication use, genetic data or existing comorbidities. Further collaborative efforts that has already been started [34] by researchers and clinicians, will facilitate this research.

ACKNOWLEDGMENT

The authors hereby thank Ajay Verma for his advisory role and support, the research team at ABM including S. Waninger, G. Rupp, M. McConnell, S. Smith, and C. Richard for data collection and technical discussions. David Salat (Massachusetts General Hospital), Joanne Hamilton (U of California San Diego), Bradley Boeve and Erik K. St Louis (Mayo Clinic), for their advisory role, and facilitating patient recruitment. This work was partially supported by National Institute of Health (R44AG050326, R44AG054256) and Biogen Inc.

REFERENCES

[1] J. W. Britton et al., Electroencephalography (EEG): An Introductory Text and Atlas of Normal and Abnormal Findings in Adults, Children, and Infants. Chicago: American Epilepsy Society, 2016.

[2] F. Vecchio et al., "Chapter 15 - Resting state cortical EEG rhythms in Alzheimer's disease: toward EEG markers for clinical applications: a review," in Supplements to Clinical Neurophysiology, vol. 62, E. Başar, C. Başar-Eroĝlu, A. Özerdem, P. M. Rossini, and G. G. Yener, Eds. Elsevier, 2013, pp. 223–236.

[3] R. Wang, J. Wang, H. Yu, X. Wei, C. Yang, and B. Deng, "Power spectral density and coherence analysis of Alzheimer's EEG," Cogn Neurodyn, vol. 9, no. 3, pp. 291–304, Jun. 2015.

[4] S. Waninger et al., "Event-related potentials during sustained attention and memory tasks: Utility as biomarkers for mild cognitive impairment," Alzheimers Dement (Amst), vol. 10, pp. 452–460, 2018.

[5] C. Babiloni et al., "Abnormal cortical sources of resting state electroencephalographic rhythms in single treatment-naïve HIV individuals: A statistical z-score index," Clin Neurophysiol, vol. 127, no. 3, pp. 1803–1812, Mar. 2016.

[6] A. Horvath, A. Szucs, G. Csukly, A. Sakovics, G. Stefanics, and A. Kamondi, "EEG and ERP biomarkers of Alzheimer's disease: a critical review," Front Biosci (Landmark Ed), vol. 23, pp. 183–220, Jan. 2018.

[7] C. Berka et al., "Mapping the trajectory of neurodegenerative disease with neurophysiological biomarkers acquired during sleep and waking," presented at the The 14th International Conference on Alzheimer's & Parkinson's Diseases, Lisbon, Portugal, 26-Mar-2019.

[8] C. Micanovic and S. Pal, "The diagnostic utility of EEG in early-onset dementia: a systematic review of the literature with narrative analysis," J Neural Transm (Vienna), vol. 121, no. 1, pp. 59–69, Jan. 2014.

[9] A. Tsolaki, D. Kazis, I. Kompatsiaris, V. Kosmidou, and M. Tsolaki, "Electroencephalogram and Alzheimer's Disease: Clinical and Research Approaches," International Journal of Alzheimer's Disease, 2014. .

[10] S. Smith, "EEG in neurological conditions other than epilepsy: when does it help, what does it add?," J Neurol Neurosurg Psychiatry, vol. 76, no. Suppl 2, pp. ii8–ii12, Jun. 2005.

[11] M. Nuwer, "Assessment of digital EEG, quantitative EEG, and EEG brain mapping: Report of the American Academy of Neurology and the American Clinical Neurophysiology Society*," Neurology, vol. 49, no. 1, p. 277, Jul. 1997.

[12] K. L. Coburn, E. C. Lauterbach, N. N. Boutros, K. J. Black, D. B. Arciniegas, and C. E. Coffey, "The Value of Quantitative Electroencephalography in Clinical Psychiatry: A Report by the Committee on Research of the American Neuropsychiatric Association," JNP, vol. 18, no. 4, pp. 460–500, Oct. 2006.

[13] R. W. Thatcher, "Validity and Reliability of Quantitative Electroencephalography," Journal of Neurotherapy, vol. 14, no. 2, pp. 122–152, May 2010.

[14] R. Cassani, M. Estarellas, R. San-Martin, F. J. Fraga, and T. H. Falk, "Systematic Review on Resting-State EEG for Alzheimer's Disease Diagnosis and Progression Assessment," Disease Markers, 2018.

[15] C. R. Jack et al., "NIA-AA Research Framework: Toward a biological definition of Alzheimer's disease," Alzheimer's & Dementia, vol. 14, no. 4, pp. 535–562, Apr. 2018.

[16] A. Delorme and S. Makeig, "EEGLAB: an open source toolbox for analysis of single-trial EEG dynamics including independent component analysis," J. Neurosci. Methods, vol. 134, no. 1, pp. 9–21, Mar. 2004.

[17] A. Mognon, J. Jovicich, L. Bruzzone, and M. Buiatti, "ADJUST: An automatic EEG artifact detector based on the joint use of spatial and temporal features," Psychophysiology, vol. 48, no. 2, pp. 229–240, 2011.

[18] L. Pion-Tonachini, S. Makeig, and K. Kreutz-Delgado, "Crowd labeling latent Dirichlet allocation," Knowl Inf Syst, vol. 53, no. 3, pp. 749–765, Dec. 2017.

[19] S. M. Bowyer, "Coherence a measure of the brain networks: past and present," Neuropsychiatric Electrophysiology, vol. 2, no. 1, p. 1, Jan. 2016.

[20] R. Srinivasan, W. R. Winter, J. Ding, and P. L. Nunez, "EEG and MEG coherence: measures of functional connectivity at distinct spatial scales of neocortical dynamics," J Neurosci Methods, vol. 166, no. 1, pp. 41–52, Oct. 2007.

[21] J. Dauwels, F. Vialatte, T. Musha, and A. Cichocki, "A comparative study of synchrony measures for the early diagnosis of Alzheimer's disease based on EEG," Neuroimage, vol. 49, no. 1, pp. 668–693, Jan. 2010.

[22] E. Stomrud, O. Hansson, L. Minthon, K. Blennow, I. Rosén, and E. Londos, "Slowing of EEG correlates with CSF biomarkers and reduced cognitive speed in elderly with normal cognition over 4 years," Neurobiol. Aging, vol. 31, no. 2, pp. 215–223, Feb. 2010.

[23] Garces,Pilar ; Hipp,Joerg , Meghdadi, Amir H., Waninger,Shani ; (2), Anne Smith ; Boak, Lauren ; Schobel, Scott, "Resting state electroencephalography (EEG) biomarkers in Huntington's disease (HD): Feasibility, reliability and relation to HD pathology," presented at the 14th Annual HD Therapeutics Conference, Palm Springs, CA, USA, 2019.

[24] E. Neto, F. Biessmann, H. Aurlien, H. Nordby, and T. Eichele, "Regularized Linear Discriminant Analysis of EEG Features in Dementia Patients," Front. Aging Neurosci., vol. 8, 2016.

[25] Y. Chen et al., "DCCA cross-correlation coefficients reveals the change of both synchronization and oscillation in EEG of Alzheimer disease patients," Physica A: Statistical Mechanics and its Applications, vol. 490, pp. 171–184, Jan. 2018.

[26] M. G. Knyazeva et al., "Topography of EEG multivariate phase synchronization in early Alzheimer's disease," Neurobiology of Aging, vol. 31, no. 7, pp. 1132–1144, Jul. 2010.

[27] F.-B. Vialatte, J. Dauwels, M. Maurice, T. Musha, and A. Cichocki, "Improving the Specificity of EEG for Diagnosing Alzheimer's Disease," International Journal of Alzheimer's Disease, 2011.

[28] M. Buscema et al., "An improved I-FAST system for the diagnosis of Alzheimer's disease from unprocessed electroencephalograms by using robust invariant features," Artificial Intelligence in Medicine, vol. 64, no. 1, pp. 59–74, May 2015.

[29] P. Raamana, L. Wang, H. Rosen, B. Miller, M. Weiner, and M. F. Beg, "Differential diagnosis among Alzheimer's disease, frontotemporal disease and healthy aging: Comparative study using subcortical features," Alzheimer's & Dementia: The Journal of the Alzheimer's Association, vol. 8, no. 4, pp. P163–P164, Jul. 2012.

[30] J. N. Caviness et al., "Cortical Phosphorylated α-Synuclein Levels Correlate with Brain Wave Spectra in Parkinson's Disease," Mov Disord, vol. 31, no. 7, pp. 1012–1019, Jul. 2016.

[31] M. S. Albert et al., "The diagnosis of mild cognitive impairment due to Alzheimer's disease: Recommendations from the National Institute on Aging-Alzheimer's Association workgroups on diagnostic guidelines for Alzheimer's disease," Alzheimer's & Dementia: The Journal of the Alzheimer's Association, vol. 7, no. 3, pp. 270–279, May 2011.

[32] O. F. F. Odish, K. Johnsen, P. van Someren, R. A. C. Roos, and J. G. van Dijk, "EEG may serve as a biomarker in Huntington's disease using machine learning automatic classification," Scientific Reports, vol. 8, no. 1, p. 16090, Oct. 2018.

[33] G. M. McKhann et al., "The diagnosis of dementia due to Alzheimer's disease: Recommendations from the National Institute on Aging-Alzheimer's Association workgroups on diagnostic guidelines for Alzheimer's disease," Alzheimer's & Dementia: The Journal of the Alzheimer's Association, vol. 7, no. 3, pp. 263–269, May 2011.

[34] A. W. Toga and K. L. Crawford, "The Alzheimer's Disease Neuroimaging Initiative Informatics Core: A Decade in Review," Alzheimers Dement, vol. 11, no. 7, pp. 832–839, Jul. 2015.
